# Evolutionary Diversification of the Maize Str-like Gene Family Revealed Through Sequence, Structural and Functional Analyses

**DOI:** 10.3390/genes17070774

**Published:** 2026-06-30

**Authors:** Xiaowei Liu, Lanping Gu, Chengming Zhang, Jie Li, Kun Cai, Kehao Cui, Zhuoling Zhong, Huiming Qiu, Yi Zhang, Yongming Liu

**Affiliations:** 1 Chengdu Agricultural College, Chengdu 611130, China; liuxw@cdnkxy.edu.cn (X.L.); 19908196749@139.com (L.G.); 17760390563@163.com (J.L.); 18800811773@139.com (K.C.); 15756346223@163.com (K.C.); 18011625869@163.com (Z.Z.); 1623378132@163.com (H.Q.); 2639249410@163.com (Y.Z.); 2 College of Chemistry and Life Sciences, Chengdu Normal University, Chengdu 611130, China; zhangcm@cndu.edu.cn; 3 Yazhouwan National Laboratory, Sanya 572025, China

**Keywords:** maize, STR-like genes, evolutionary constraint, codon usage bias, protein structural variation, GO enrichment, tissue-specific expression

## Abstract

Strictosidine synthases (STRs) are catalytic enzymes involved in terpenoid indole alkaloid biosynthesis, whereas STR-like (STRL) genes in cereal crops remain poorly understood. Previous studies of the maize STR-like (STRL) gene family have mainly provided genome-wide identification, phylogenetic classification, structural annotation and expression profiling, but the evolutionary constraints and molecular mechanisms underlying STRL diversification remain insufficiently resolved. In this study, we investigated the maize STRL gene family from an evolutionary and structural perspective by integrating sequence divergence, codon usage bias, selection pressure, protein structural modelling, Gene Ontology (GO) enrichment and tissue-specific expression analysis. A total of 21 *ZmSTRL* genes were analyzed and their comparative and phylogenetic analyses revealed conserved lineages together with maize-associated expansion patterns. Codon usage and neutrality analyses indicated heterogeneous evolutionary constraints among *ZmSTRL* genes, suggesting that mutational pressure alone does not explain their sequence divergence. Protein conservation and three-dimensional structural modelling showed a generally conserved STR-related catalytic framework, while member-specific variation in terminal and loop regions suggested localized structural divergence. GO enrichment supported conserved catalytic and metabolic signatures, but these associations were interpreted as putative functional evidence rather than direct functional confirmation. Tissue-specific qRT-PCR analysis revealed divergent expression patterns among selected *ZmSTRL* genes in root, stem, leaf, and anther tissues, indicating possible regulatory specialization. Overall, this study provides an evolutionary-constraint-based framework for understanding STRL diversification in maize and identifies candidate genes and structural features for future functional validation.

## 1. Introduction

Plant secondary metabolism plays critical roles in growth, development and adaptation to environmental stresses [1,2]. Among these metabolites, terpenoid indole alkaloids (TIAs) represent a large and structurally diverse group of natural products with important physiological and pharmacological functions [3,4]. Strictosidine synthases (STRs) are key catalytic enzymes in TIA biosynthesis, catalyzing the condensation of tryptamine and secologanin to form strictosidine, a central precursor for downstream alkaloid production [5,6,7]. In plants, STRs serve as a precursor for the biosynthesis of a wide range of alkaloids, with more than 2000 compounds derived from this molecule [8]. Although STR enzymes have been extensively characterized in alkaloid-producing plants, increasing genomic evidence has revealed the presence of STR-like (STRL) homologs in non-specialized plant lineages, including cereal crops, where their biological roles and evolutionary diversification remain poorly understood [7].

Despite the identification of STRL gene families in multiple plant species, including Arabidopsis and rice, most previous studies have primarily focused on gene identification, phylogenetic classification, gene structure, conserved motif and expression profiling [9,10,11]. In maize, Gu et al. (2023) systematically characterized the STRL gene family and provided important information regarding chromosomal distribution, phylogenetic relationships, conserved motifs, cis-regulatory elements, tissue-specific expression (not RT-PCR validated) and stress-responsive expression patterns. These studies established a valuable foundation for understanding the maize STRL gene family. However, they primarily provided descriptive characterization and offered limited insight into the evolutionary mechanisms responsible for sequence divergence, structural diversification and regulatory specialization within the STRL family. In particular, the evolutionary constraints shaping codon usage patterns, sequence conservation, protein structural variation and transcriptional divergence among maize STRL genes remain largely unresolved [12].

With the rapid expansion of plant genomic resources, integrative gene family analyses have become increasingly important for understanding the evolutionary history and functional diversification of plant gene families [13,14,15]. Gene family expansion is frequently driven by duplication events followed by sequence divergence and differential selective constraints acting at both nucleotide and protein levels [16,17,18]. Codon usage bias reflects the combined influence of mutational pressure and natural selection, providing valuable information on the evolutionary optimization of coding sequences. Likewise, analyses of sequence conservation and protein structural variation can reveal how conserved catalytic frameworks are maintained while allowing localized divergence that may contribute to functional differentiation. Furthermore, tissue-specific expression patterns provide evidence for regulatory divergence following gene duplication [19,20]. Nevertheless, few studies have integrated these complementary evolutionary, structural, and regulatory analyses to investigate the diversification of the maize STRL gene family [21,22].

In this study, we hypothesize that diversification of the maize STRL gene family has been shaped by heterogeneous evolutionary constraints acting on coding sequence composition, protein structural organization and transcriptional regulation. To test this hypothesis, we integrated codon usage bias analysis, neutrality analysis, sequence conservation, protein structural modelling, GO enrichment and tissue-specific expression profiling to investigate the evolutionary diversification of maize STRL genes. Building upon previous descriptive genome-wide characterizations, this study establishes an evolutionary constraint-based framework for understanding how sequence variation, structural conservation, and regulatory divergence have collectively contributed to STRL gene diversification in maize and provides candidate genes for future functional investigation. 

## 2. Materials and Methods

### 2.1. Plant Materials

The maize (*Zea mays* L.) inbred line B73 was used as the plant material in this study. Surface-sterilized seeds were germinated and grown in nutrient soil under controlled greenhouse conditions with a 16 h light/8 h dark photoperiod at 25 °C and 70% humidity. Plants were maintained under uniform growth conditions to ensure synchronized development. For tissue-specific expression analysis, maize plants were grown to the appropriate developmental stage at which reproductive tissues were fully formed. Leaf, stem, root and anther tissues were collected at the corresponding developmental stages. Anther samples were harvested from developing tassels at the reproductive stage when anther formation was complete. All collected samples were immediately flash-frozen in liquid nitrogen after harvesting and stored at −80 °C until further molecular analysis. 

### 2.2. Identification and Characterization of ZmSTRL Genes in Maize

Genome-wide sequence resources for maize and rice were acquired from the Ensembl Plant/Gramene databases (https://ensembl.gramene.org/; Access Date: 23 October 2025). The reference STR/STRL protein sequences used for candidate identification were obtained from rice (*Oryza sativa*) and *A. thaliana*. To identify STR-related proteins in maize, previously reported rice STR protein sequences were used as references for similarity search against the maize protein dataset. BLASTP analyses were conducted using version 2.10.0+ and an *E*-value threshold of 1 × 10^−6^ was used together with a minimum sequence identity of 50%. Duplicate and redundant candidate sequences were removed by comparing protein sequences and gene identifiers and only non-redundant sequences containing the conserved STR domain were retained for subsequent analyses. The initially obtained maize candidates were further examined for domain confirmation. For this purpose, HMMER (v3.3.2) analysis was carried out using the STR-domain hidden Markov model profile from Pfam (PF03088). Only sequences containing the conserved STR domain were retained as putative members of the STR gene family. The confirmed STR proteins were then subjected to physicochemical characterization. Protein lengths, molecular weights, theoretical isoelectric point, predicted subcellular localizations and grand average of hydropathicity values were determined using the ExPASy ProtParam server (http://web.expasy.org/protparam/; Access Date: 22 November 2025). The detailed information regarding the reference STR/STRL sequences and the *O. sativa* and *A. thaliana* accession numbers is provided in Tables S5–S7. 

### 2.3. Comparative Genomic Organization and Evolutionary Analysis of STR/STRL Genes

Chromosomal locations of *STR/STRL* genes in *Zea mays* were retrieved from genome annotation files used in this study. For comparative analysis, orthologous *STR/STRL* gene locus information for *Oryza sativa* (Japonica/Nipponbare) and *Arabidopsis thaliana* (Col-0) was obtained from publicly accessible genome databases (EnsemblPlants and NCBI Genome database; Access Date: 13 April 2026) using validated gene identifiers and locus annotations. Genes were mapped to their respective chromosomes based on genomic coordinate positions (start and end sites) extracted from *GFF3* annotation files. To quantify chromosomal distribution patterns, gene frequency per chromosome was calculated for each species and normalized according to chromosome length where applicable to avoid bias due to chromosome size variation. Species-wise chromosomal distribution matrices were constructed and used to evaluate conservation and divergence of *STR/STRL* genomic organization across monocot and dicot lineages. Comparative visualization of chromosomal gene distribution was performed using Python (v3.9), employing Pandas (v1.5.3) for data processing, NumPy (v1.24.3) for matrix construction and Matplotlib/Seaborn (v3.7.1) for heatmap generation. Schematic chromosomal mapping was additionally generated using the MG2C tool (v2.1) and custom Python plotting scripts to illustrate gene positioning across chromosomes. Phylogenetic relationships were inferred based on multiple sequence alignment of STR coding sequences generated using ClustalW (v2.1) and MUSCLE (v3.8.31) algorithms. Evolutionary trees were constructed using MEGA X software with the Neighbor-Joining method and 1000 bootstrap replicates to assess clade robustness. Furthermore, chromosomal distribution, phylogeny, gene structure and cis-regulatory analyses of *ZmSTRL* genes were performed as described in Supplementary Methods S1–S3. 

### 2.4. Sequence Evolution, Codon Usage Bias, Structural Conservation and Functional Divergence Analysis

Codon usage indices, including the Effective Number of Codons (ENC), GC content at the third codon position (GC3) and GC content at the first and second codon positions (GC12), were calculated using CodonW (v1.4.4) and custom Python scripts. Neutrality plot analysis (GC12 vs. GC3) was performed to assess the relative contribution of mutational pressure and natural selection on codon usage patterns. Visualization of codon bias distributions and neutrality plots was carried out using Matplotlib and Seaborn. Pairwise sequence similarity among STR genes was computed using BLASTp/BLASTn (NCBI BLAST+ suite) followed by construction of a similarity matrix based on percentage identity scores. The resulting matrix was visualized as a heatmap using the Python Seaborn package. This analysis was used to evaluate intra-family divergence patterns and identify closely and distantly related STR members. Protein sequence alignment of STR family members was performed using Clustal Omega (v1.2.4) and conservation profiles were generated to assess positional amino acid conservation across the gene family. Conservation scores were calculated based on alignment variability and visualized to identify highly conserved functional regions and variable domains. Biochemical properties of STR proteins, including isoelectric point (pI) and molecular weight (MW), were computed using the ExPASy ProtParam tool (Access Date: 10 May 2026). These parameters were used to evaluate physicochemical diversity among STR proteins and infer potential functional differentiation. 

### 2.5. Three-Dimensional Structural Modelling of ZmSTRL Protein

Three-dimensional structures of *ZmSTRL* proteins were predicted by homology modelling using the SWISS-MODEL server (https://swissmodel.expasy.org/interactive; Access Date: 18 June 2025) [23]. The resulting models were examined to evaluate overall structural conservation and potential structural divergence among family members. The stereochemical reliability and overall quality of the predicted protein models were assessed through Ramachandran plot analysis.

### 2.6. Gene Ontology Enrichment and Functional Annotation Analysis

Gene Ontology (GO) annotation of the *ZmSTRL* gene family was performed using the agriGO platform (http://bioinfo.cau.edu.cn/agriGO/index.php Access Date: 13 July 2025) [24]. GO terms were classified into the three standard categories of biological process, cellular component and molecular function and GO terms with *p* < 0.05 were considered significantly enriched. To obtain a more comprehensive functional overview of the *ZmSTRL* gene family, enrichment results were further visualized using the OmicShare online platform (http://www.omicshare.com/tools Access Date: 16 July 2025). Bar plots and bubble plots were generated to display the most enriched GO terms and their significance levels. Hierarchical GO relationship networks were constructed to examine the functional associations among enriched categories.

### 2.7. Tissue-Specific Expression Analysis of ZmSTRL Genes

Gene-specific primers for selected *ZmSTRL* genes were designed using NCBI Primers-BLAST (https://www.ncbi.nlm.nih.gov/tools/primer-blast/ Access Date: 11 December 2025). Primer pairs were designed to specifically amplify the target genes and primer information is provided in Table S8. Tissue samples, including roots, stems, leaves and anthers, were collected from the maize inbred line B73 at an appropriate developmental stage.

Total RNA was extracted using TRIzol reagents according to a previously described protocol [25]. For each tissue type, three biological replicates were included. Each biological replicate was analyzed with three technical replicates. First-strand cDNA was synthesized using PrimeScript RT kits, followed by quantitative real-time PCR (qRT-PCR) (CFX96 Real-Time PCR Detection System, Bio-Rad Laboratories, Hercules, CA, USA) analysis to determine the expression level of selected *ZmSTRL* genes. The maize *ZmActin* gene (Zm00001d010159) was used as an internal reference for normalization. The primer sequences for *ZmActin* were 5′-*TCACCCTGTGCTGCTGACCG*-3′ and 5′-*GAACCGTGTGGCTAACCCA*-3′. Relative expression levels were calculated using the 2^−ΔΔCt^ method [25] and are presented as mean ± standard error. Statistical analysis was performed using GraphPad Prism version 9.0 and differences among tissues were evaluated using ANOVA. The tissue-specific expression patterns were subsequently compared among organs to evaluate potential functional specialization of *ZmSTRL* genes in maize. In addition to qRT-PCR-based validation, expression profiles of *ZmSTRL* gene family members were retrieved from publicly available transcriptomic databases, including MaizeGDB qTeller (B73v4 reference genome) and MaizeMine. Normalized RNA-seq expression data across root, stem, leaf and anther tissues were extracted and integrated to complement the experimental expression analysis.

## 3. Results

### 3.1. Genome-Wide Identifications and Structural–Evolutionary Overview of ZmSTRL Genes

A total of 21 STR-like genes, designated *ZmSTRL1*–*ZmSTRL21*, were identified in the maize genome based on conserved STR-domain searches and sequence homology. All predicted *ZmSTRL* proteins retained the conserved strictosidine synthase-related domain, supporting their classification as members of the STRL family. Basic physicochemical characterization revealed variation in protein length, molecular weight, isoelectric point, hydropathicity, and predicted subcellular localization, indicating molecular heterogeneity among family members (Table S1). To avoid redundancy with previous genome-wide characterization of the maize SSL/STRL family, detailed analyses of chromosomal distribution, gene structure, conserved motifs, promoter cis-elements and basic phylogenetic grouping are provided in the Supplementary Materials (Figures S1–S3). These analyses confirmed non-random chromosomal distribution, conservation of core STR-related motifs, and lineage-associated clustering among STRL genes. Together, these background analyses provided the structural and genomic basis for subsequent investigation of codon usage bias, sequence divergence, protein structural variation, GO enrichment and tissue-specific expression patterns.

### 3.2. Comparative Genomic Organization Supports Lineage-Associated STRL Clustering

**Figure 1:**
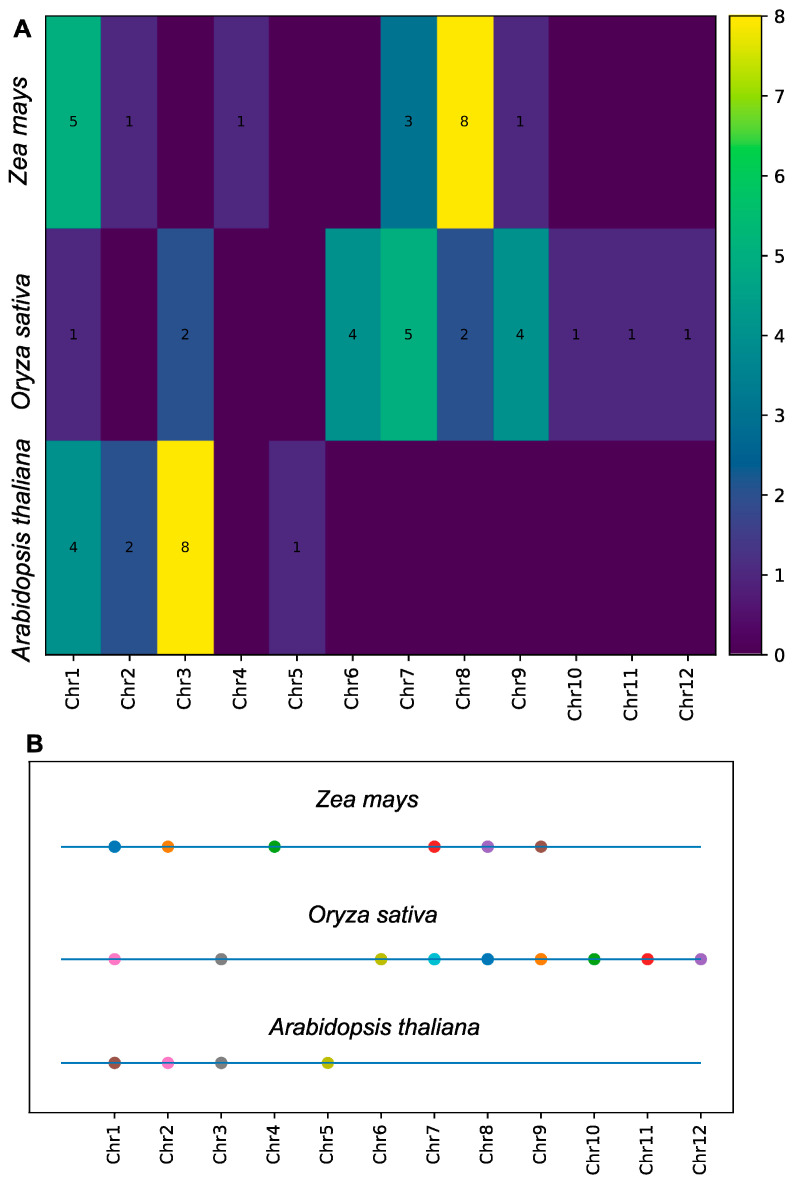
Comparative chromosomal organization of STR/STRL genes in maize, rice and Arabidopsis. (**A**) Heatmap showing the distribution of STR/STRL genes across chromosomes in three plant species. (**B**) Schematic representation of STR/STRL gene positions along chromosomes. Each dot represents an individual STR/STRL gene, highlighting species-specific clustering and distribution patterns across genomes.

Comparative chromosomal mapping of STR/STRL genes in maize, rice and Arabidopsis revealed lineage-associated differences in genomic organization. In maize, *ZmSTRL* genes showed a non-random distribution, with a prominent cluster on chromosome 8, suggesting localized expansion of the family. By contrast, rice STR/STRL genes were more broadly distributed across chromosomes, whereas Arabidopsis showed a distinct clustering pattern on chromosome 3. These species-specific distribution patterns indicate that STRL genomic organization has been shaped by lineage-dependent expansion and dispersion events (Figure 1A,B). Because chromosomal distribution of maize SSL/STRL genes has been reported previously, the detailed chromosome maps are presented in Figure S1 and this analysis is used here only as genomic context for the evolutionary constraint and divergence analyses.

### 3.3. Codon Usage Bias and Selection Pressure Reveal Differential Evolutionary Constraints Among ZmSTRL Genes

**Figure 2:**
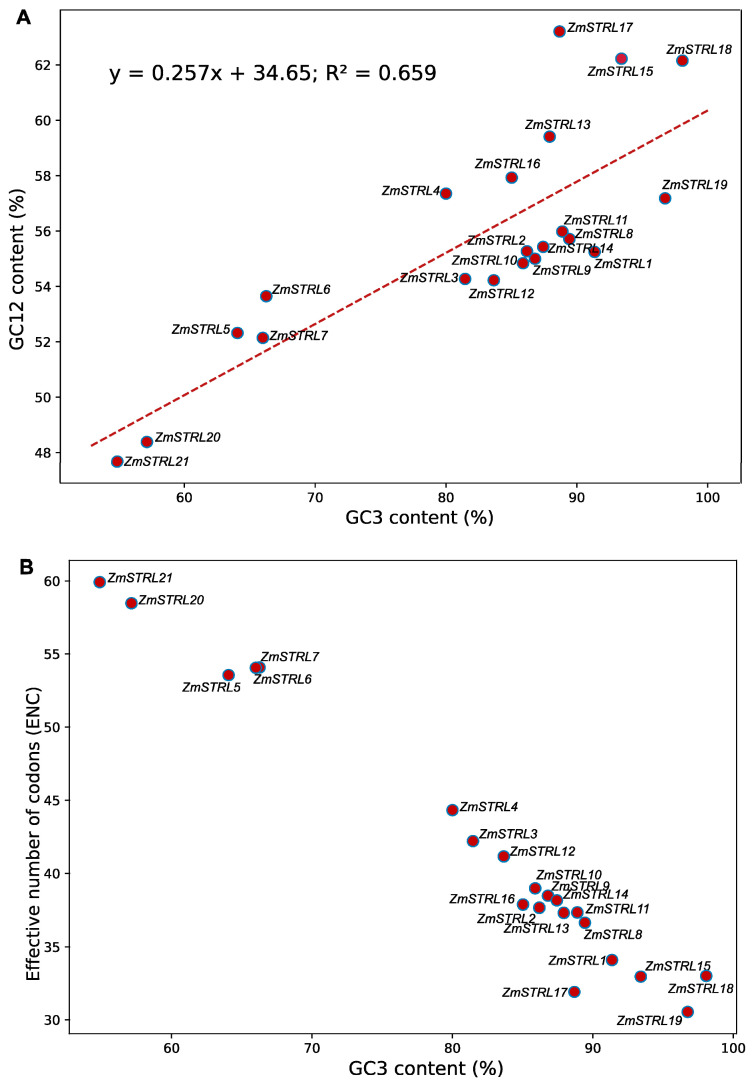
**Codon usage bias and selection pressure analysis of STR genes.** (**A**) Relationship between effective number of codons (ENC) and GC content at third codon position (GC3), illustrating codon usage bias across STR genes. (**B**) Neutrality analysis showing the relationship between GC12 and GC3 values, indicating the relative contributions of mutational pressure and natural selection in shaping codon usage patterns.

To investigate the evolutionary forces shaping *ZmSTRL* gene diversification, codon usage bias and neutrality analyses were performed. Effective number of codons (ENC) and GC content at the third codon position (GC3) showed moderate variation among *ZmSTRL* genes (Figure 2A), indicating differences in synonymous codon usage across family members. These differences suggest that coding sequences have experienced varying evolutionary pressures during diversification. Neutrality analysis (GC12 vs. GC3) was further performed to evaluate the relative contributions of mutational pressure and natural selection (Figure 2B). The weak correlation observed between GC12 and GC3 indicates that codon usage patterns cannot be explained solely by mutational pressure. Instead, the deviation from the neutrality expectation suggests that natural selection has contributed to shaping codon usage patterns in *ZmSTRL* genes, although the relative influence may differ among individual family members. Overall, these findings indicate that *ZmSTRL* genes have evolved under differential selective constraints rather than uniform neutral evolution. This sequence-level heterogeneity provides additional evidence that diversification of the maize STRL family has involved multiple evolutionary processes acting on coding sequence composition.

### 3.4. Sequence Divergence and Protein Conservation Reveal Heterogeneous Evolutionary Trajectories of ZmSTRL Genes

**Figure 3:**
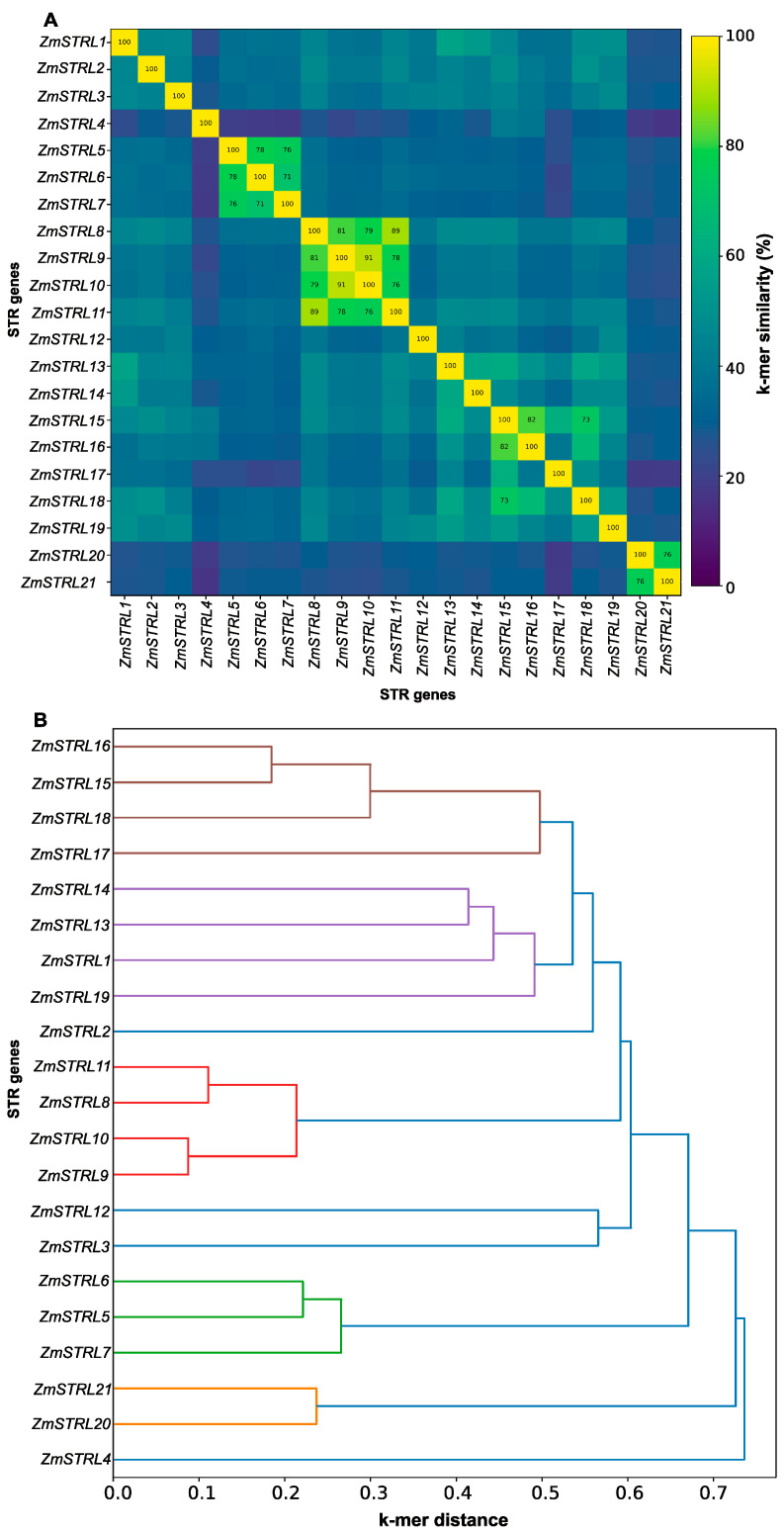
**Evolutionary relationships and divergence patterns of STR genes.** (**A**) Pairwise sequence similarity heatmap of STR genes, showing heterogeneous similarity distribution and identification of highly conserved and divergent gene pairs. (**B**) Phylogenetic tree illustrating evolutionary relationships among STR genes, revealing distinct clades and lineage-specific expansion patterns.

**Figure 4:**
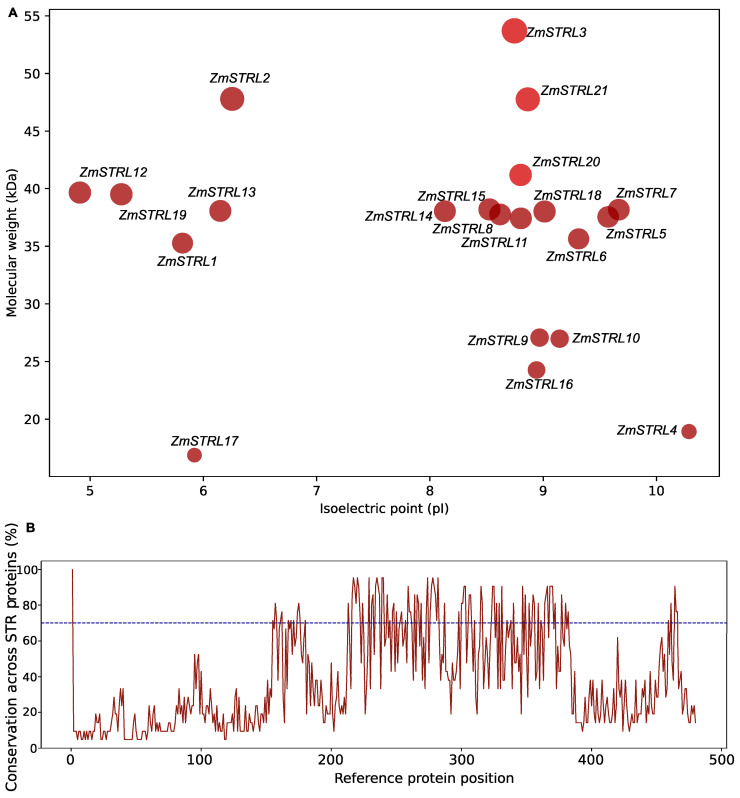
**Structural conservation and biochemical properties of STR proteins.** (**A**) Position-wise conservation profile of STR proteins showing conserved central domains and variable terminal regions, indicating functional constraints and flexible regions. (**B**) Distribution of STR proteins based on pI and MW, demonstrating biochemical diversity within the gene family.

To further investigate evolutionary diversification within the *ZmSTRL* family, pairwise sequence similarity and protein conservation analyses were performed. Pairwise sequence similarity revealed substantial variation among *ZmSTRL* genes, with several gene pairs showing high sequence conservation, whereas others displayed markedly lower similarity (Figure 3A). These differences indicate unequal levels of sequence divergence among family members, suggesting that individual *ZmSTRL* genes have experienced distinct evolutionary histories following gene duplication. Phylogenetic analysis grouped *ZmSTRL* genes into several well-supported clades (Figure 3B), providing a general framework for evolutionary classification. Consistent with the sequence similarity analysis, closely related genes were generally clustered within the same phylogenetic groups, whereas more divergent members occupied separate lineages. These relationships support lineage-associated diversification within the maize STRL family while serving primarily as a framework for comparative evolutionary interpretation. Protein conservation analysis further demonstrated that the central STR-related catalytic region remained highly conserved across *ZmSTRL* proteins, whereas the N- and C-terminal regions exhibited greater sequence variability (Figure 4A). This pattern suggests that strong evolutionary constraints have preserved the catalytic core while permitting localized divergence in peripheral regions. Consistent with this observation, variation in molecular weight and isoelectric point among *ZmSTRL* proteins (Figure 4B) indicates diversification in physicochemical properties that may influence protein stability or molecular interactions. These results indicate that *ZmSTRL* genes retain a conserved catalytic framework while exhibiting differential sequence and protein-level divergence, supporting heterogeneous evolutionary trajectories across the gene family. 

### 3.5. Predicted Three-Dimensional Structures Reveal Conserved Catalytic Architecture with Localized Structural Divergence

**Figure 5:**
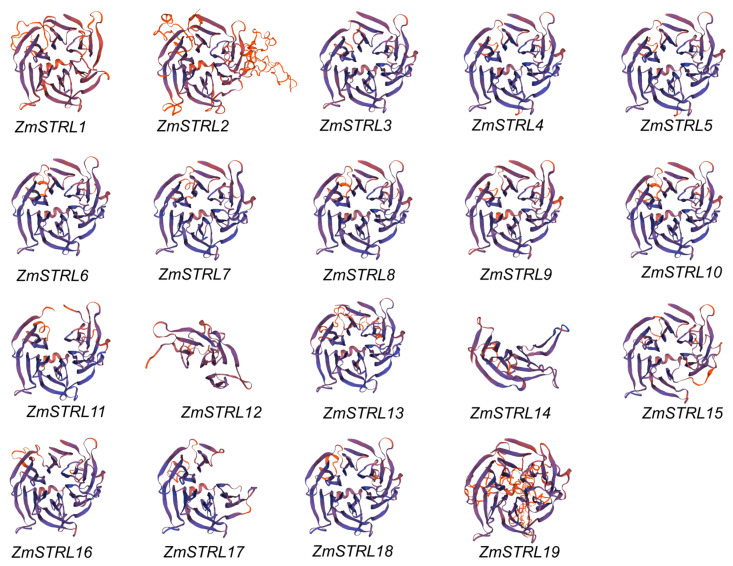
**Predicted three-dimensional structures of *ZmSTRL* proteins.** Predicted 3D models of *ZmSTRL1*–*ZmSTRL19* showing structural variations, including simplified folds and extended loop regions in a subset of proteins, are highlighted, indicating potential functional diversification within the maize STRL family.

Predicted three-dimensional models demonstrated that *ZmSTRL* proteins share a highly conserved overall structural architecture characterized by the canonical β-propeller fold typical of the strictosidine synthase family (Figure 5). The conservation of this structural framework across most *ZmSTRL* members suggests that the catalytic core has been maintained under strong evolutionary constraint. Despite this overall structural conservation, localized variation was observed among several *ZmSTRL* proteins. Differences in loop regions and peripheral structural elements were evident in a subset of proteins, whereas the central β-propeller scaffold remained largely unchanged. These observations indicate that structural divergence has occurred primarily outside the conserved catalytic framework. The coexistence of a highly conserved catalytic core with localized structural variability is consistent with the sequence divergence and protein conservation patterns described above. Together, these results suggest that while the fundamental structural architecture of *ZmSTRL* proteins has been evolutionarily conserved, limited structural modifications may contribute to molecular diversification among individual family members. However, the potential functional significance of these structural differences requires future experimental validation. 

### 3.6. GO Enrichment Supports Putative Catalytic and Metabolic Functional Signatures of ZmSTRL Genes 

**Figure 6:**
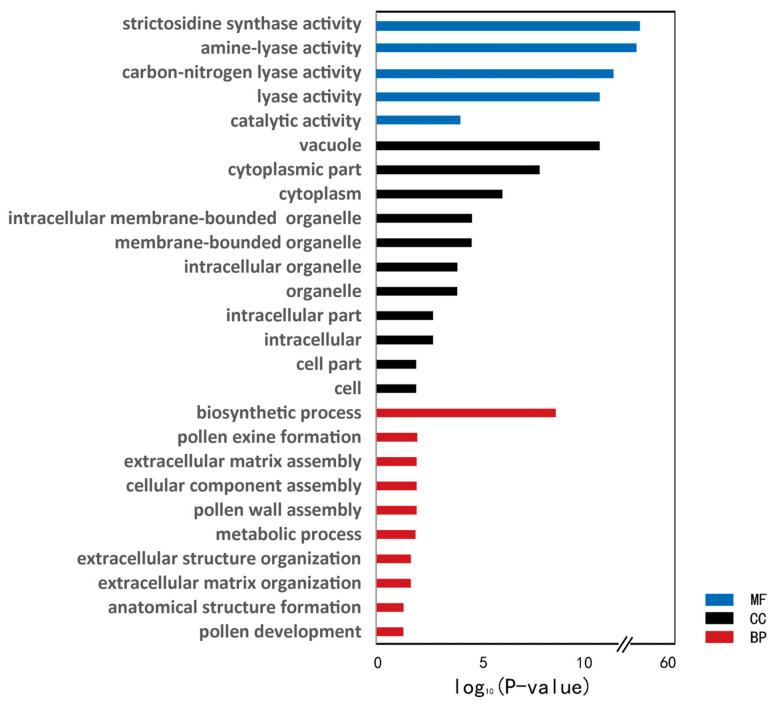
**GO enrichment analysis of *ZmSTRL* gene family.** GO enrichment analysis was performed to classify *ZmSTRL* genes into three main categories. The enriched GO terms are displayed based on significance (*p* < 0.05), highlighting the involvement of *ZmSTRL* genes in catalytic activity, biosynthetic processes and cellular components. BP: Biological Process, CC: Cellular Component, MF: Molecular Function.

**Figure 7:**
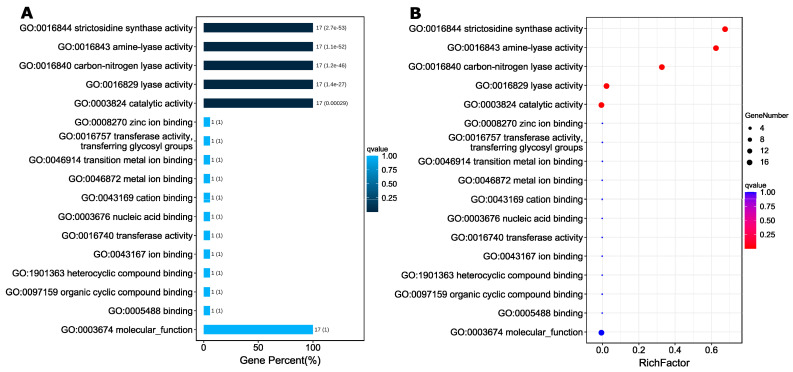
**Functional annotation patterns of *ZmSTRL* genes based on enriched GO terms.** (**A**) Significantly enriched GO terms associated with *ZmSTRL* genes. (**B**) The distribution and significance of the same enriched GO terms. Bubble size represents the number of *ZmSTRL* genes assigned to each term, while color indicates enrichment significance based on q-value.

GO enrichment analysis was performed to infer the potential functional context of *ZmSTRL* genes across molecular function, cellular component and biological process categories (Figure 6 and Figure 7). Overall, the enrichment profile indicated that *ZmSTRL* genes are predominantly associated with catalytic enzyme activity and intracellular metabolic processes. 

In the molecular function category, enriched terms included strictosidine synthase activity, amine-lyase activity and carbon–nitrogen lyase activity (Figure 6). These terms support the conserved enzymatic identity of the *ZmSTRL* family and are consistent with the conserved STR-related domain and predicted β-propeller structural framework. Hierarchical GO classification further showed that these terms were grouped within broader lyase-related functional categories (Figure S4, Table S2), suggesting that *ZmSTRL* proteins share a conserved catalytic annotation profile.

In the cellular component category, enriched terms such as cytoplasm, cytoplasmic part and vacuole suggested that *ZmSTRL* proteins are associated mainly with intracellular compartments (Figure 6). The hierarchical structure of these terms (Figure S5, Table S3) further indicates their localization within intracellular and organelle-associated environments, consistent with their predicted metabolic roles. In the biological process category, biosynthetic process and metabolic process were the dominant enriched terms, while lower-frequency terms related to reproductive development, including pollen exine formation, pollen wall assembly and extracellular structure organization, were also detected (Figure 6). These developmental terms involved fewer genes and should therefore be interpreted cautiously as putative functional associations. GO hierarchy analysis (Figure S6, Table S4) indicates that these terms are embedded within broader metabolic and cellular process networks. Overall, GO enrichment supports a conserved catalytic and metabolic annotation profile for the *ZmSTRL* family, while suggesting possible functional divergence among a subset of genes (Figure 7A,B). However, because GO enrichment is based on computational annotation, these results should be considered hypothesis-generating evidence and require future experimental validation.

### 3.7. Tissue-Specific Expression Patterns Indicate Regulatory Divergence Among ZmSTRL Genes

**Figure 8:**
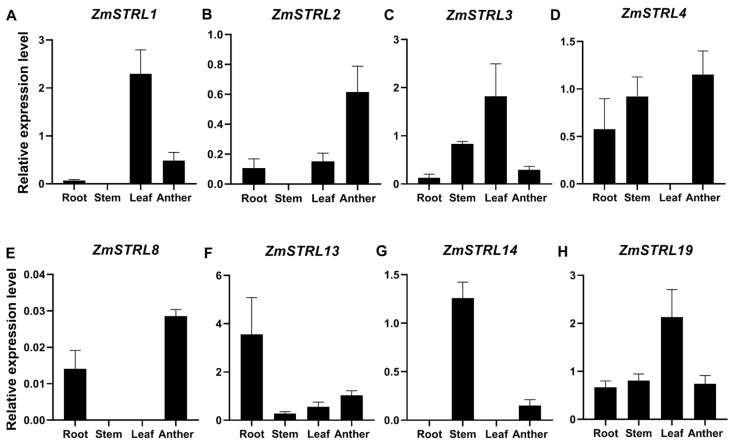
**Tissue-specific expression profile of selected *ZmSTRL* genes in maize.** (**A**–**H**) Relative expression levels of selected *ZmSTRL* genes were analyzed in root, stem, leaf and anther tissues. Data are presented as mean ± SE.

To investigate transcriptional divergence within the *ZmSTRL* gene family, expression profiles of selected genes were analyzed across root, stem, leaf, and anther tissues using qRT-PCR (Figure 8). The analyzed genes showed distinct tissue-dependent expression patterns, indicating heterogeneous transcriptional regulation among family members. Some genes, including *ZmSTRL3*, *ZmSTRL13* and *ZmSTRL19*, were expressed across all examined tissues, suggesting relatively broad transcriptional activity. In contrast, other genes showed more tissue-restricted expression patterns, with *ZmSTRL2* and *ZmSTRL4* showing very low or undetectable expression in stem and leaf tissues, respectively. Several genes also exhibited tissue-preferential expression, including higher expression of *ZmSTRL1* in leaves, *ZmSTRL14* in stems and *ZmSTRL8* in anthers. The elevated expression of *ZmSTRL8* in anther tissue is consistent with the reproductive-associated GO terms detected in the enrichment analysis. However, this relationship should be interpreted as a putative functional association rather than direct evidence of reproductive function. Furthermore, expression profiles of additional *ZmSTRL* genes (*ZmSTRL*5–6, *ZmSTRL*9–12, *ZmSTRL*15–18 and *ZmSTRL*20–21) indicate that the *ZmSTRL* gene family exhibits highly heterogeneous and tissue-specific transcriptional regulation across root, stem, leaf, and anther tissues (Figure S7). Overall, the tissue-specific qRT-PCR results suggest regulatory divergence among *ZmSTRL* genes and support the broader model that gene family diversification in maize involves both sequence-level evolutionary constraints and transcriptional specialization.

## 4. Discussion

Strictosidine synthases play central roles in terpenoid indole alkaloid biosynthesis by catalyzing a key step linking primary and secondary metabolism [7,10]. While STR family members have been extensively characterized in medicinal plants, their homologs in cereal crops remain less understood [26,27]. In this context, the present study provides an integrative evolutionary analysis of the STR-like (STRL) gene family in maize by combining sequence-level, structural, regulatory and expression-based evidence to infer patterns of molecular diversification. 

Genome-wide analysis indicates that STRL genes in maize exhibit non-random chromosomal distribution and lineage-specific expansion patterns [28,29,30,31]. The clustering of multiple *ZmSTRL* genes on chromosome 8 suggests localized duplication-associated expansion, consistent with evolutionary hotspots observed in other plant gene families [32,33]. Phylogenetic reconstruction further provides a general classification framework supporting the coexistence of conserved ancestral lineages and monocot-associated divergence [34,35,36,37]. However, because the phylogenetic analysis was based on a Neighbor-Joining approach, broad evolutionary interpretations should be made cautiously. Importantly, sequence-level heterogeneity observed among *ZmSTRL* genes suggests that phylogenetic grouping alone is insufficient to explain their full evolutionary divergence, implying additional constraint layers acting at the coding sequence level [11,20,38,39], indicating strong constraint on the core enzymatic architecture [40,41]. However, variation in gene structure, motif distribution, and predicted protein architecture suggests that structural conservation coexists with localized diversification [42,43]. Codon usage and selection pressure analyses further indicate heterogeneous evolutionary constraints, where natural selection appears to contribute more strongly than mutational pressure to coding sequence evolution [19,44,45]. Together, these findings support a model in which STRL genes are evolutionarily constrained at the catalytic core while showing more variable constraint patterns in peripheral or divergent regions.

Protein-level analyses further reinforce this model. Conservation of the central β-propeller region suggests preservation of the catalytic framework, whereas variability in terminal regions and physicochemical properties indicates structural flexibility that may influence protein stability, interaction potential, or regulatory behavior [10]. Sequence similarity and phylogenetic divergence patterns collectively demonstrate the coexistence of highly conserved and more divergent gene pairs, reflecting differential evolutionary pressures acting across the gene family [46]. Expression profiling further indicates transcriptional divergence among *ZmSTRL* members, suggesting regulatory specialization following gene duplication events [12,20]. Importantly, GO enrichment and promoter analyses provide supporting evidence for catalytic identity and potential regulatory responsiveness, although these results remain predictive and require experimental validation.

GO enrichment and tissue-specific expression analyses provide complementary but supportive evidence for the functional context of *ZmSTRL* genes. GO enrichment consistently indicates that *ZmSTRL* proteins are associated with conserved catalytic and lyase-related functions, supporting their predicted enzymatic identity. However, these annotations are predictive in nature and primarily reflect computational inference rather than direct functional validation [12,47]. Similarly, tissue-specific expression patterns suggest transcriptional diversification among *ZmSTRL* members, with both broadly expressed and tissue-preferential genes indicating regulatory divergence following gene duplication. While these patterns are consistent with potential functional specialization, they should be interpreted as preliminary evidence of regulatory differentiation rather than definitive functional assignment [10,12]. 

Importantly, previous studies on STRL genes, including genome-wide characterization efforts such as Gu et al. (2023), have primarily focused on gene identification, phylogenetic classification, structural annotation, and expression profiling, thereby providing a valuable descriptive catalog of family members [12]. In contrast, the present study builds on this foundation by integrating sequence-level evolutionary constraints, codon usage bias, structural variation, GO-based putative functional annotation, and expression divergence to infer a constraint-based evolutionary model of STRL diversification in maize. Collectively, our results support a unified framework in which STRL gene family diversification is associated with heterogeneous selective pressures acting at multiple molecular levels. Conserved catalytic architecture appears to be maintained under strong structural constraint, whereas sequence, structural, and regulatory variation may contribute to progressive molecular diversification among paralogs. This study therefore shifts STRL gene analysis from descriptive genome-wide cataloging toward an evolutionary constraint-based understanding of gene family diversification in maize.

## 5. Conclusions

This study provides an integrative evolutionary framework for understanding the STR-like (*ZmSTRL*) gene family in maize by combining sequence-level, structural, and regulatory analyses. Rather than focusing solely on descriptive genome-wide characterization, our analyses demonstrate that *ZmSTRL* genes exhibit heterogeneous evolutionary constraints reflected in codon usage bias, sequence divergence, and protein structural variation while maintaining a highly conserved STR-related catalytic framework. GO enrichment and tissue-specific expression analyses further provide supportive evidence for regulatory diversification and putative functional differentiation among family members, although these computational and expression-based predictions require future experimental validation. Collectively, our findings suggest that the evolution of the maize STRL gene family has been shaped by the interplay between strong conservation of the catalytic core and progressive diversification at the sequence, structural, and regulatory levels. This study establishes a constraint-based perspective for understanding STRL gene family evolution in maize and provides candidate genes and hypotheses for future functional investigation.

## Data Availability

The original contributions presented in this study are included in the article/Supplementary Materials. Further inquiries can be directed to the corresponding author(s).

## Supplementary Materials

The following supporting information can be downloaded at: https://www.mdpi.com/article/10.3390/genes17070774/s1, Table S1. Genome-wide detection and basic features of the STR gene family in maize (*Z. mays*), Figure S1. Phylogenetic relationships of STR proteins from maize, rice and Arabidopsis, Figure S2. Cis-regulatory element distribution in the promoter regions of *ZmSTRL* genes, Figure S3. Gene structure, conserved domains, motif composition and chromosomal distribution of *ZmSTRL* genes, Figure S4. Molecular function hierarchy of enriched GO terms for *ZmSTRL* genes, Figure S5. Cellular component hierarchy of enriched GO terms for *ZmSTRL* genes, Figure S6. Biological process hierarchy of enriched GO terms for *ZmSTRL* genes, Figure S7. Tissue-specific expression analysis of *ZmSTRL* gene family in maize, Table S2. GO enrichment statistics for *ZmSTRL* gene family belonging to Molecular Functions, Table S3. GO enrichment statistics for *ZmSTRL* gene family belonging to Cellular Component, Table S4. GO enrichment statistics for *ZmSTRL* gene family belonging to Biological Process, Table. S5. The accession numbers of *Oryza sativa*, Table S6. The accession numbers of *Arabidopsis thaliana*, Table S7. The detailed information of the reference STR/STRL sequences, Method S1. Chromosomal distribution of *ZmSTRL* genes, Method S2. Cis-regulatory element analysis of *ZmSTRL* promoters, Method S3. Phylogenetic analysis, gene structure and conserved motif characterization, Table S8. Forward (*F*) and reverse (*R*) primer sequences used in real-time quantitative PCR. Refs. [48,49,50] are cited in supplementary files.

## Abbreviations

The following abbreviations are used in this manuscript:

STR	Strictosidine synthase
GRAVY	Grand average of hydropathicity
HMM	Hidden Markov model
GSDS	Gene Structure Display Server
TSS	Transcription start site
ABRE	Abscisic acid-responsive element
UTR	Untranslated regions
ARE	Anaerobic induction element
