# Supplementary material for: Evolutionary Diversification of the Maize Str-like Gene Family Revealed Through Sequence, Structural and Functional Analyses
Source: Genes (Basel). 2026 Jun 30;17(7):774. doi: 10.3390/genes17070774 (PMC13410046; doi:10.3390/genes17070774)
Supplement: Supplementary file 1 [file genes-17-00774-s001.zip › genes-4408145-supplementary.pdf]

Supporting Information

Evolutionary diversification of the maize STR-like gene family revealed through sequence, structural and functional analyses

The following supplementary information available for this article.

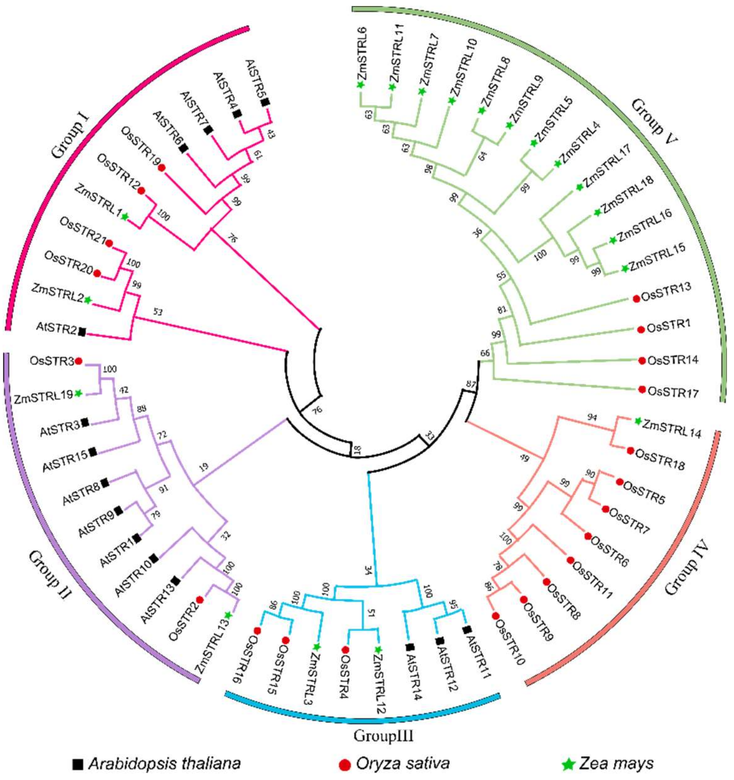

Figure S1. Phylogenetic relationships of STR proteins from maize, rice and Arabidopsis. The phylogenetic tree was generated using the complete amino acid sequences of STR proteins from maize (*Z. mays*), rice (*O. sativa*) and *A. thaliana*. The STR proteins were classified into five distinct groups (Groups I–V), indicated by different colors. The clustering pattern highlights both evolutionary relationships and subgroup-specific expansions, revealing conserved and maize-specific diversification within the *ZmSTRL* gene family.

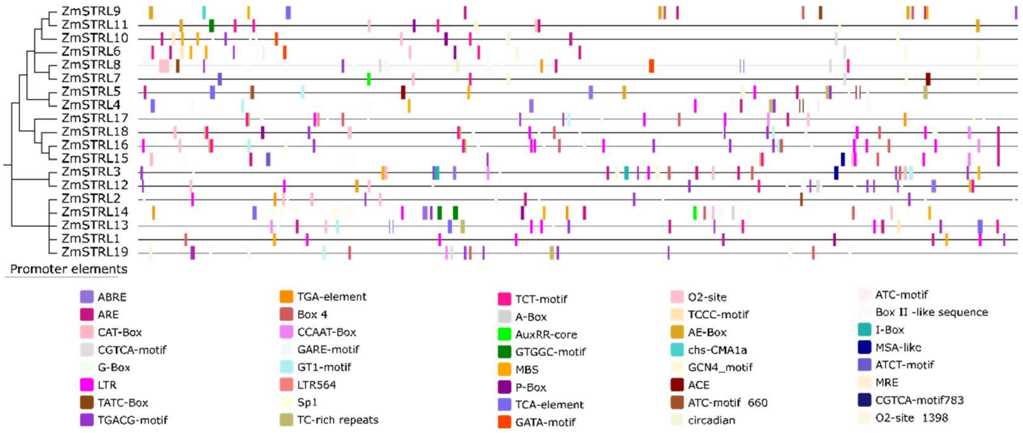

Figure S2. Cis-regulatory element distribution in the promoter regions of *ZmSTRL* genes. Different colored boxes indicate distinct promoter elements associated with hormone responsiveness, abiotic stress responses, light regulation and developmental processes.

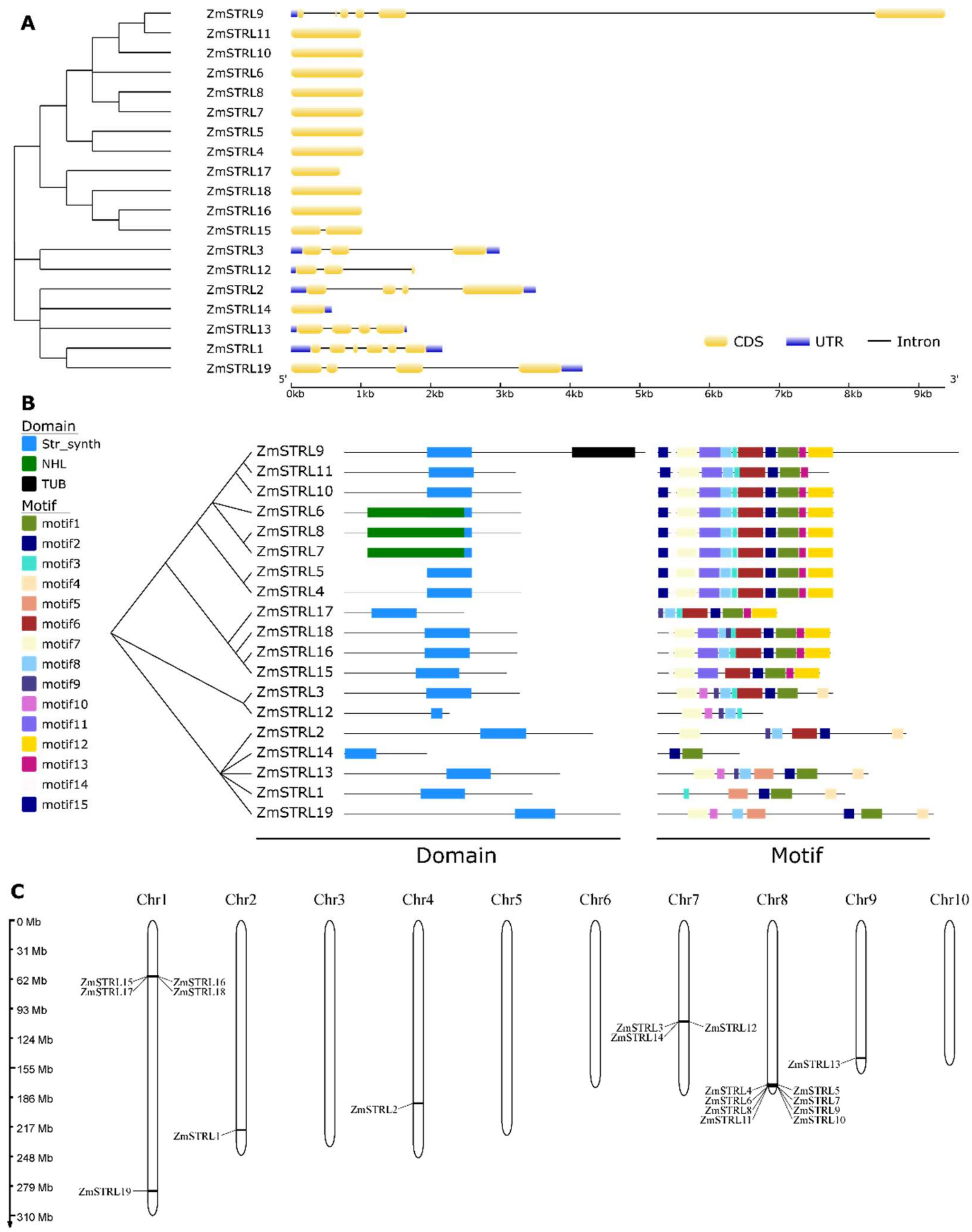

Figure S3. Gene structure, conserved domains, motif composition and chromosomal distribution of *ZmSTRL* genes. (**A**) Exon–intron organization of *ZmSTRL* genes. Yellow boxes represent coding sequences (CDS), blue boxes indicate untranslated regions (UTRs), and black lines denote introns. (**B**) Conserved domain and motif organization of *ZmSTRL* proteins. Different colored boxes represent distinct conserved motifs within each protein sequence. (**C**) Chromosomal localization of *ZmSTRL* genes on maize chromosomes according to the B73 reference genome.

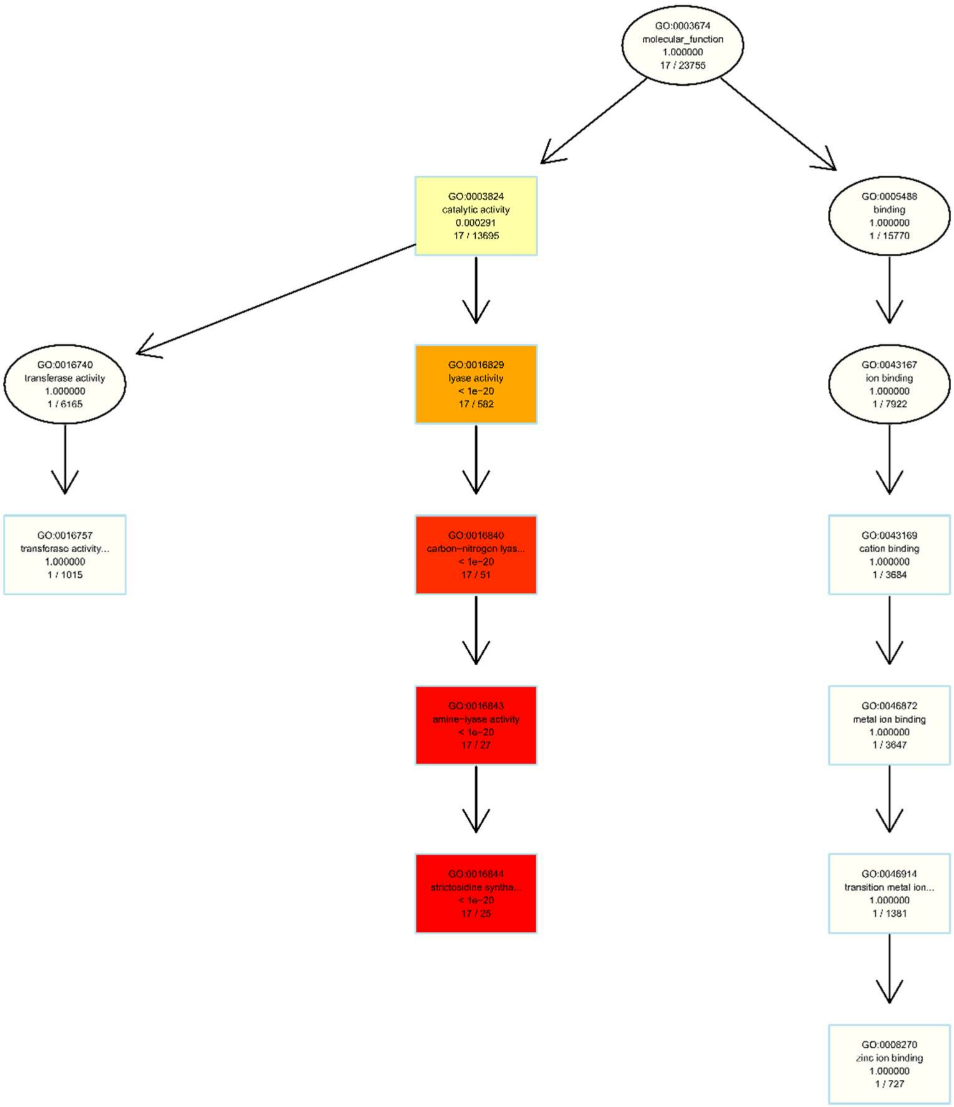

Figure S4. Molecular function hierarchy of enriched GO terms for *ZmSTRL* genes. Hierarchical analysis emphasizes the relationships among catalytic functions, including strictosidine synthase activity, amine-lyase activity, carbon–nitrogen lyase activity, lyase activity and overall catalytic activity. The network shows gene distribution and functional clustering of molecular function terms.

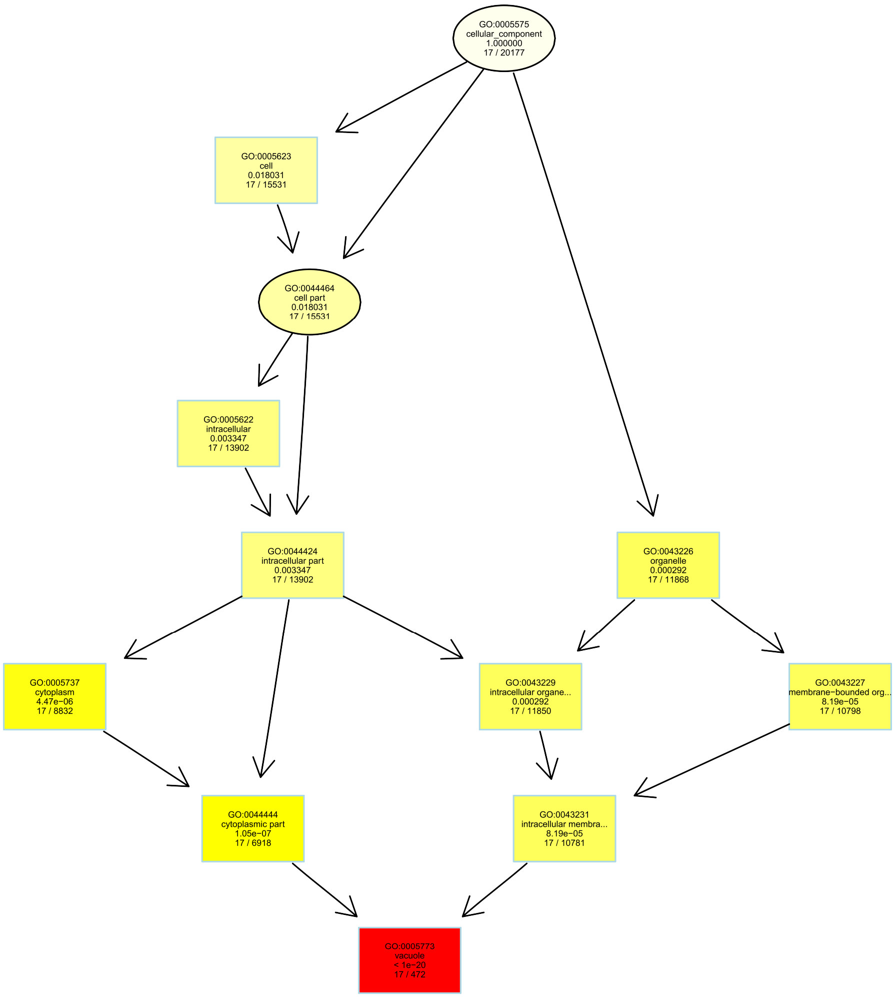

Figure S5. Cellular component hierarchy of enriched GO terms for *ZmSTRL* genes. The hierarchical network illustrates the relationships among intracellular compartments, including vacuole, cytoplasmic part, cytoplasm, intracellular organelle and membrane-bounded organelle. Its highlights cellular localization patterns for the *ZmSTRL* family.

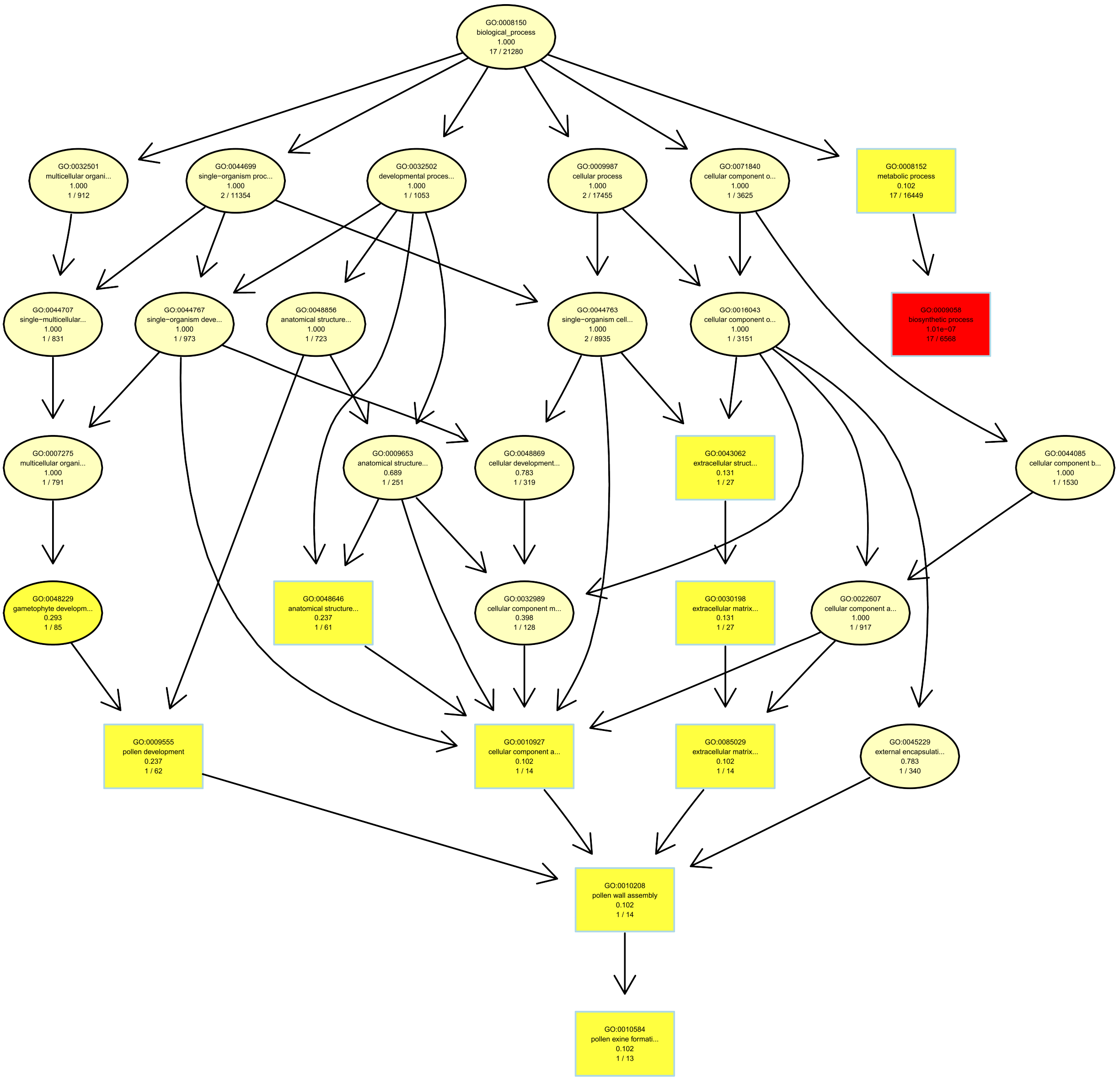

Figure S6. Biological process hierarchy of enriched GO terms for *ZmSTRL* genes. The hierarchical network highlights biosynthetic process, metabolic process and selected development-related terms, such as pollen exine formation, pollen wall assembly and extracellular matrix organization. Its provides detailed insight into the functional roles of *ZmSTRL* genes in maize development and metabolism.

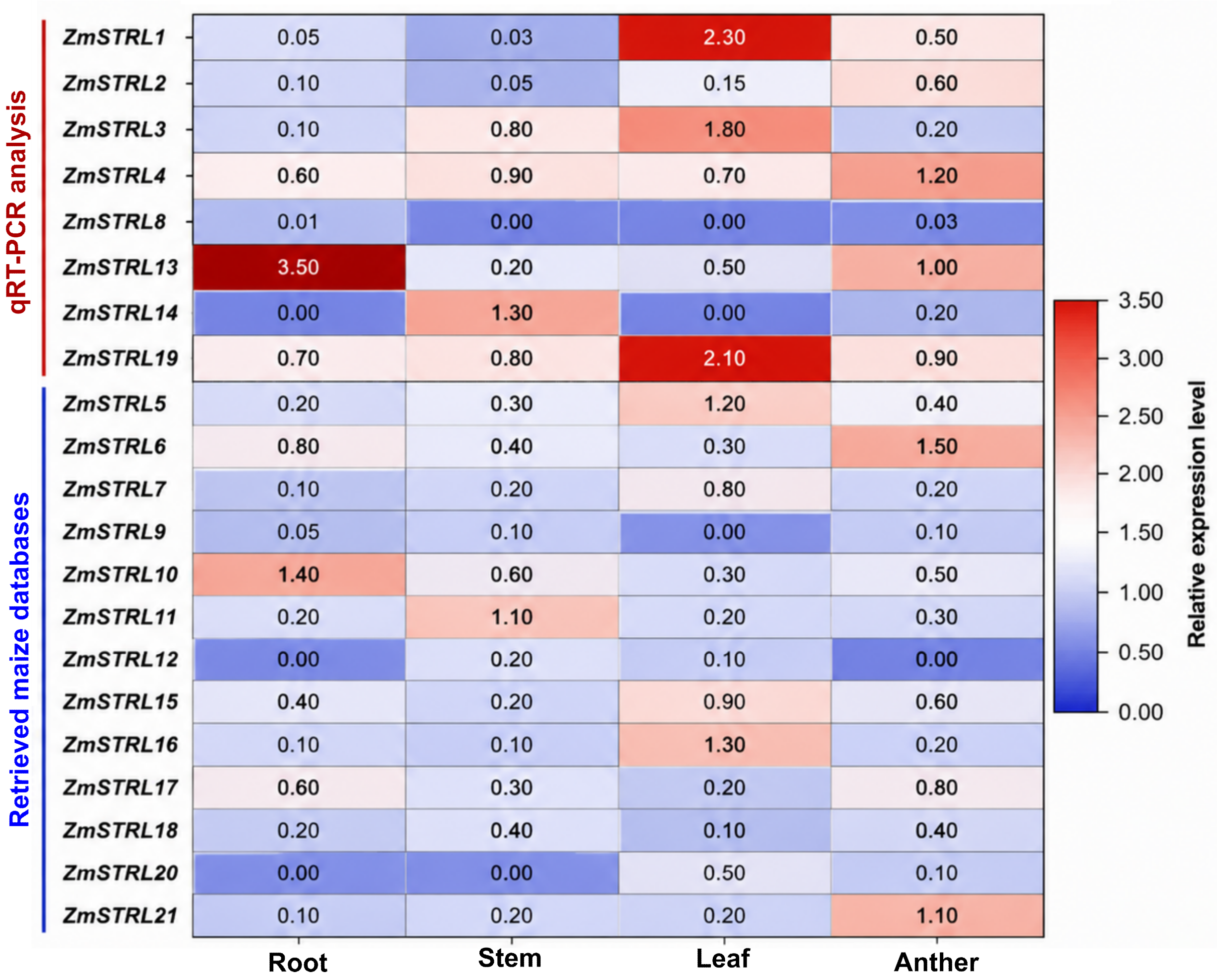

Figure S7. Tissue-specific expression analysis of *ZmSTRL* gene family in maize. Expression analysis of *ZmSTRL* genes across root, stem, leaf and anther tissues was performed using publicly available RNA-seq datasets retrieved from MaizeGDB qTeller and MaizeMine. Expression values represent normalized transcript abundance across tissues. Red indicates higher expression and blue indicates lower expression.

Table. S1 Genome-wide detection and basic features of the STR gene family in maize (*Z. mays*).

Gene name	Gene_id	Chr.	Gene length (bp)	CDS (bp)	Size (aa)	Exon No.	Strand	Subcellular localization	Protein characteristic		
									pl	MW (kDa)	GRAVY
ZmSTRL1	Zm00001d006853	Chr2	2165	1104	367	6	+	chloroplast	5.25	39.46	0.05
ZmSTRL2	Zm00001d052471	Chr4	3511	1461	486	4	+	extracellular	8.67	51.56	-0.07
ZmSTRL3	Zm00001d034032	Chr1	4182	1620	539	4	+	Plasma membrane	8.86	59.22	-0.29
ZmSTRL4	Zm00001d012249	Chr8	1038	1038	345	1	-	chloroplast	6	36.66	-0.03
ZmSTRL5	Zm00001d012252	Chr8	1038	1038	345	1	-	chloroplast	5.81	36.63	-0.05
ZmSTRL6	Zm00001d012366	Chr8	1038	1038	345	1	-	chloroplast	6.61	36.74	-0.06
ZmSTRL7	Zm00001d012368	Chr8	1038	1038	345	1	-	chloroplast	6.61	36.81	-0.05
ZmSTRL8	Zm00001d012371	Chr8	1038	1038	345	1	-	chloroplast	7.02	36.78	-0.02
ZmSTRL9	Zm00001d012373	Chr8	9380	1767	588	6	-	chloroplast	9.55	64.12	-0.14
ZmSTRL10	Zm00001d012377	Chr8	1038	1038	345	1	-	chloroplast	7.01	36.75	-0.04
ZmSTRL11	Zm00001d012375	Chr8	1005	1008	335	1	-	chloroplast	6.6	35.54	-0.08
ZmSTRL12	Zm00001d020307	Chr7	1772	621	206	3	+	chloroplast	8.86	22.24	-0.08
ZmSTRL13	Zm00001d047858	Chr9	1657	1239	412	4	+	Endoplasmic reticulum	6	46.54	-0.30
ZmSTRL14	Zm00001d020308	Chr7	581	486	161	1	+	cytoplasmic	6.83	16.98	0.06
ZmSTRL15	Zm00001d029108	Chr1	1024	954	317	2	+	extracellular	6.96	34.11	-0.01
ZmSTRL16	Zm00001d029111	Chr1	1017	1017	338	1	+	extracellular	6.08	36.31	0.00
ZmSTRL17	Zm00001d029115	Chr1	705	705	234	1	+	chloroplast	5.38	25.38	-0.11
ZmSTRL18	Zm00001d029117	Chr1	1017	1017	338	1	+	extracellular	6.08	36.33	0.01
ZmSTRL19	Zm00001d020306	Chr7	2991	1032	343	3	-	chloroplast	7.73	36.17	0.01

Chr. indicates chromosome location. CDS (bp) indicates coding sequence length. Size (aa) denotes the number of amino acids in the encoded protein. pI represents the theoretical isoelectric point. MW (kDa) represents molecular weight. GRAVY indicates the grand average of hydropathicity. Strand indicates coding strand orientation.

Table S2. GO enrichment statistics for *ZmSTRL* genes family belong to Molecular Functions.

#	GO ID	Description	out (17)	All (23755)	P value	p. adjust
1	GO:0016844	strictosidine synthase activity	17 (100%)	25 (0.11%)	0.000000	**0.000000**
2	GO:0016843	amine-lyase activity	17 (100%)	27 (0.11%)	0.000000	**0.000000**
3	GO:0016840	carbon-nitrogen lyase activity	17 (100%)	51 (0.21%)	0.000000	**0.000000**
4	GO:0016829	lyase activity	17 (100%)	582 (2.45%)	0.000000	**0.000000**
5	GO:0003824	catalytic activity	17 (100%)	13695 (57.65%)	0.000085	**0.000291**
6	GO:0008270	zinc ion binding	1 (5.88%)	727 (3.06%)	0.410557	1.000000
7	GO:0016757	transferase activity, transferring glycosyl groups	1 (5.88%)	1015 (4.27%)	0.524127	1.000000
8	GO:0046914	transition metal ion binding	1 (5.88%)	1381 (5.81%)	0.638879	1.000000
9	GO:0046872	metal ion binding	1 (5.88%)	3647 (15.35%)	0.941253	1.000000
10	GO:0043169	cation binding	1 (5.88%)	3684 (15.51%)	0.943065	1.000000
11	GO:0003676	nucleic acid binding	1 (5.88%)	5297 (22.3%)	0.986304	1.000000
12	GO:0016740	transferase activity	1 (5.88%)	6165 (25.95%)	0.993963	1.000000
13	GO:0043167	ion binding	1 (5.88%)	7922 (33.35%)	0.998992	1.000000
14	GO:1901363	heterocyclic compound binding	1 (5.88%)	10044 (42.28%)	0.999913	1.000000
15	GO:0097159	organic cyclic compound binding	1 (5.88%)	10064 (42.37%)	0.999915	1.000000
16	GO:0005488	binding	1 (5.88%)	15770 (66.39%)	1.000000	1.000000
17	GO:0003674	molecular_function	17 (100%)	23755 (100%)	1.000000	1.000000

p-value is calculated using Fisher's exact test for enrichment; adjusted p-value is corrected for multiple testing using the Benjamini-Hochberg method.

Table S3. GO enrichment statistics for *ZmSTRL* genes family belong to Cellular Component.

#	GO ID	Description	out (17)	All (20177)	P value	p. adjust
1	GO:0005773	vacuole	17 (100%)	472 (2.34%)	0.000000	**0.000000**
2	GO:0044444	cytoplasmic part	17 (100%)	6918 (34.29%)	0.000000	**0.000000**
3	GO:0005737	cytoplasm	17 (100%)	8832 (43.77%)	0.000001	**0.000004**
4	GO:0043231	intracellular membrane-bounded organelle	17 (100%)	10781 (53.43%)	0.000023	**0.000082**
5	GO:0043227	membrane-bounded organelle	17 (100%)	10798 (53.52%)	0.000024	**0.000082**
6	GO:0043229	intracellular organelle	17 (100%)	11850 (58.73%)	0.000117	**0.000292**
7	GO:0043226	organelle	17 (100%)	11868 (58.82%)	0.000120	**0.000292**
8	GO:0005622	intracellular	17 (100%)	13902 (68.9%)	0.001772	**0.003347**
9	GO:0044424	intracellular part	17 (100%)	13902 (68.9%)	0.001772	**0.003347**
10	GO:0005623	cell	17 (100%)	15531 (76.97%)	0.011667	**0.018031**
11	GO:0044464	cell part	17 (100%)	15531 (76.97%)	0.011667	**0.018031**
12	GO:0005634	nucleus	1 (5.88%)	5871 (29.1%)	0.997116	1.000000

13	GO:0016021	integral component of membrane	2 (11.76%)	8084 (40.07%)	0.997952	1.000000
14	GO:0031224	intrinsic component of membrane	2 (11.76%)	8202 (40.65%)	0.998228	1.000000
15	GO:0044425	membrane part	2 (11.76%)	8712 (43.18%)	0.999070	1.000000
16	GO:0016020	membrane	2 (11.76%)	9885 (48.99%)	0.999815	1.000000
17	GO:0005575	cellular_component	17 (100%)	20177 (100%)	1.000000	1.000000

p-value is calculated using Fisher's exact test for enrichment; adjusted p-value is corrected for multiple testing using the Benjamini-Hochberg method.

Table S4. GO enrichment statistics for *ZmSTRL* genes family belong to Biological Process.

#	GO ID	Description	out (17)	All (21280)	P value	p. adjust
1	GO:0009058	biosynthetic process	17 (100%)	6568 (30.86%)	0.000000	**0.000000**
2	GO:0010584	pollen exine formation	1 (5.88%)	13 (0.06%)	0.010339	0.102349
3	GO:0010208	pollen wall assembly	1 (5.88%)	14 (0.07%)	0.011130	0.102349
4	GO:0010927	cellular component assembly involved in morphogenesis	1 (5.88%)	14 (0.07%)	0.011130	0.102349
5	GO:0085029	extracellular matrix assembly	1 (5.88%)	14 (0.07%)	0.011130	0.102349
6	GO:0008152	metabolic process	17 (100%)	16449 (77.3%)	0.012533	0.102349
7	GO:0030198	extracellular matrix organization	1 (5.88%)	27 (0.13%)	0.021360	0.130830
8	GO:0043062	extracellular structure organization	1 (5.88%)	27 (0.13%)	0.021360	0.130830
9	GO:0048646	anatomical structure formation involved in morphogenesis	1 (5.88%)	61 (0.29%)	0.047647	0.237209
10	GO:0009555	pollen development	1 (5.88%)	62 (0.29%)	0.048410	0.237209
11	GO:0048229	gametophyte development	1 (5.88%)	85 (0.4%)	0.065801	0.293113
12	GO:0032989	cellular component morphogenesis	1 (5.88%)	128 (0.6%)	0.097515	0.398186
13	GO:0009653	anatomical structure morphogenesis	1 (5.88%)	251 (1.18%)	0.182730	0.688750
14	GO:0048869	cellular developmental process	1 (5.88%)	319 (1.5%)	0.226529	0.782701
15	GO:0045229	external encapsulating structure organization	1 (5.88%)	340 (1.6%)	0.239602	0.782701
16	GO:0006281	DNA repair	1 (5.88%)	689 (3.24%)	0.428645	1.000000
17	GO:0006974	cellular response to DNA damage stimulus	1 (5.88%)	716 (3.36%)	0.441253	1.000000
18	GO:0048856	anatomical structure development	1 (5.88%)	723 (3.4%)	0.444479	1.000000
19	GO:0007275	multicellular organism development	1 (5.88%)	791 (3.72%)	0.474917	1.000000
20	GO:0044707	single-multicellular organism process	1 (5.88%)	831 (3.91%)	0.492081	1.000000
21	GO:0032501	multicellular organismal process	1 (5.88%)	912 (4.29%)	0.525233	1.000000

22	GO:0022607	cellular component assembly	1 (5.88%)	917 (4.31%)	0.527211	1.000000
23	GO:0006259	DNA metabolic process	1 (5.88%)	934 (4.39%)	0.533879	1.000000
24	GO:0044767	single-organism developmental process	1 (5.88%)	973 (4.57%)	0.548843	1.000000
25	GO:0033554	cellular response to stress	1 (5.88%)	1036 (4.87%)	0.572065	1.000000
26	GO:0032502	developmental process	1 (5.88%)	1053 (4.95%)	0.578136	1.000000
27	GO:0044085	cellular component biogenesis	1 (5.88%)	1530 (7.19%)	0.718870	1.000000
28	GO:0006950	response to stress	1 (5.88%)	2003 (9.41%)	0.813849	1.000000
29	GO:0051716	cellular response to stimulus	1 (5.88%)	2288 (10.75%)	0.855506	1.000000
30	GO:0016043	cellular component organization	1 (5.88%)	3151 (14.81%)	0.934483	1.000000
31	GO:0050896	response to stimulus	1 (5.88%)	3615 (16.99%)	0.957845	1.000000
32	GO:0071840	cellular component organization or biogenesis	1 (5.88%)	3625 (17.03%)	0.958249	1.000000
33	GO:0090304	nucleic acid metabolic process	1 (5.88%)	5112 (24.02%)	0.990651	1.000000
34	GO:0006139	nucleobase-containing compound metabolic process	1 (5.88%)	5715 (26.86%)	0.995102	1.000000
35	GO:0044710	single-organism metabolic process	1 (5.88%)	5909 (27.77%)	0.996043	1.000000
36	GO:0046483	heterocycle metabolic process	1 (5.88%)	6006 (28.22%)	0.996447	1.000000
37	GO:0006725	cellular aromatic compound metabolic process	1 (5.88%)	6106 (28.69%)	0.996823	1.000000
38	GO:1901360	organic cyclic compound metabolic process	1 (5.88%)	6226 (29.26%)	0.997224	1.000000
39	GO:0044763	single-organism cellular process	2 (11.76%)	8935 (41.99%)	0.998734	1.000000
40	GO:0034641	cellular nitrogen compound metabolic process	1 (5.88%)	7132 (33.52%)	0.999034	1.000000
41	GO:0006807	nitrogen compound metabolic process	1 (5.88%)	7542 (35.44%)	0.999414	1.000000
42	GO:0044699	single-organism process	2 (11.76%)	11354 (53.36%)	0.999952	1.000000
43	GO:0044260	cellular macromolecule metabolic process	1 (5.88%)	10379 (48.77%)	0.999989	1.000000
44	GO:0043170	macromolecule metabolic process	1 (5.88%)	11287 (53.04%)	0.999997	1.000000
45	GO:0044237	cellular metabolic process	1 (5.88%)	13653 (64.16%)	1.000000	1.000000
46	GO:0044238	primary metabolic process	1 (5.88%)	13664 (64.21%)	1.000000	1.000000
47	GO:0071704	organic substance metabolic process	1 (5.88%)	14297 (67.19%)	1.000000	1.000000
48	GO:0009987	cellular process	2 (11.76%)	17455 (82.03%)	1.000000	1.000000
49	GO:0008150	biological_process	17 (100%)	21280 (100%)	1.000000	1.000000

p-value is calculated using Fisher’s exact test for enrichment; adjusted p-value is corrected for multiple testing using the Benjamini-Hochberg method.

Table. S5. The accession numbers of *Oryza sativa*

Serial Number	Gene Name	Locus / Accession number	Species	Genome source	Chromosome
1	OsSTRL1	LOC_Os01g50330	*Oryza sativa*	Japonica / Nipponbare	Chr1
2	OsSTRL2 / OsLAP3	LOC_Os03g15710	*Oryza sativa*	Japonica / Nipponbare	Chr3
3	OsSTRL3	LOC_Os03g53950	*Oryza sativa*	Japonica / Nipponbare	Chr3
4	OsSTRL4	LOC_Os06g35950	*Oryza sativa*	Japonica / Nipponbare	Chr6
5	OsSTRL5	LOC_Os06g41820	*Oryza sativa*	Japonica / Nipponbare	Chr6
6	OsSTRL6	LOC_Os06g41830	*Oryza sativa*	Japonica / Nipponbare	Chr6
7	OsSTRL7	LOC_Os06g41850	*Oryza sativa*	Japonica / Nipponbare	Chr6
8	OsSTRL8	LOC_Os07g35970	*Oryza sativa*	Japonica / Nipponbare	Chr7
9	OsSTRL9	LOC_Os07g35990	*Oryza sativa*	Japonica / Nipponbare	Chr7
10	OsSTRL10	LOC_Os07g36040	*Oryza sativa*	Japonica / Nipponbare	Chr7
11	OsSTRL11	LOC_Os07g36060	*Oryza sativa*	Japonica / Nipponbare	Chr7
12	OsSTRL12	LOC_Os07g42250	*Oryza sativa*	Japonica / Nipponbare	Chr7
13	OsSTRL13	LOC_Os08g07810	*Oryza sativa*	Japonica / Nipponbare	Chr8
14	OsSTRL14	LOC_Os08g34330	*Oryza sativa*	Japonica / Nipponbare	Chr8
15	OsSTRL15	LOC_Os09g20684	*Oryza sativa*	Japonica / Nipponbare	Chr9
16	OsSTRL16	LOC_Os09g20700	*Oryza sativa*	Japonica / Nipponbare	Chr9
17	OsSTRL17	LOC_Os09g20720	*Oryza sativa*	Japonica / Nipponbare	Chr9
18	OsSTRL18	LOC_Os09g20810	*Oryza sativa*	Japonica / Nipponbare	Chr9

19	OsSTRL19	LOC_Os10g39710	*Oryza sativa*	Japonica / Nipponbare	Chr10
20	OsSTRL20	LOC_Os11g04660	*Oryza sativa*	Japonica / Nipponbare	Chr11
21	OsSTRL21	LOC_Os12g04424	*Oryza sativa*	Japonica / Nipponbare	Chr12

Table. S6. The accession numbers of *Arabidopsis thaliana*.

Serial Number	Gene Name	AGI locus / Accession number	Accession / UniProt	Species	Genome source
1	AtSSL1 / AtSTRL1	AT2G41300	Q9ZVB7 / F4IJZ6	*Arabidopsis thaliana*	Columbia / Col-0
2	AtSSL2 / AtSTRL2	AT2G41290	Q9SLG8	*Arabidopsis thaliana*	Columbia / Col-0
3	AtSSL3 / AtSTRL3	AT1G08470	Q8VWF6	*Arabidopsis thaliana*	Columbia / Col-0
4	AtSSL4 / AtSTRL4	AT3G51420	Q9SD07	*Arabidopsis thaliana*	Columbia / Col-0
5	AtSSL5 / AtSTRL5	AT3G51430	Q9CAZ7	*Arabidopsis thaliana*	Columbia / Col-0
6	AtSSL6 / AtSTRL6	AT3G51440	Q9SD05	*Arabidopsis thaliana*	Columbia / Col-0
7	AtSSL7 / AtSTRL7	AT3G51450	Q9SD04	*Arabidopsis thaliana*	Columbia / Col-0
8	AtSSL8 / AtSTRL8	AT3G57010	Q9M1J7	*Arabidopsis thaliana*	Columbia / Col-0
9	AtSSL9 / AtSTRL9	AT3G57020	Q9M1J6	*Arabidopsis thaliana*	Columbia / Col-0
10	AtSSL10 / AtSTRL10	AT3G57030	Q4V3D9	*Arabidopsis thaliana*	Columbia / Col-0
11	AtSSL11 / AtSTRL11	AT1G74000	P92976	*Arabidopsis thaliana*	Columbia / Col-0
12	AtSSL12 / AtSTRL12	AT1G74020	P94111	*Arabidopsis thaliana*	Columbia / Col-0
13	AtSSL13 / AtSTRL13	AT3G59530	Q9M1B4	*Arabidopsis thaliana*	Columbia / Col-0
14	AtSSL14 / AtSTRL14	AT1G74010	Q9C9C2	*Arabidopsis thaliana*	Columbia / Col-0
15	AtSSL15 / AtSTRL15	AT5G22020	Q9C586	*Arabidopsis thaliana*	Columbia / Col-0

Table S7. The detailed information of the reference STR/STRL sequences.

**>STR1**	ATGGCTCGCGATTTTACGGCGACGTTTCTTCTGCTGCTCAGCGTCGCGTCGCTGCTGG<br>TGATCTCGCCGTGCGCGGCCCAGCAGATCAAGACCACCGACACGCGGTGGAGCTAC<br>CACCTCCCGCTTCCCGACGGCGTCAGCGGCGCCGAGAGCCTCGCCTTCGACGGCAAG<br>GACGGCCTCTACACCGGCGTCTCGGACGGCCGCGTGCTCAAGTGGGGAGGCAGCGC<br>CGCCGGCTGGACCACGTTCGCGTACAATGCGAACTACCGGAAAATCCCTCTCTGCTC<br>GTCGTCCGAGGTGCCACCGGAGGAGAGGGAGAGCATCTGCGGGCGCCCGCTGGGG<br>ATCCGGTTGTTCAGGAAGACCGGCGAGCTCTACATCGCCGACGCGTACAAGGGGCT<br>GATGAAGGTCGGGCCCGACGGCGGCGAGGCCCAGGTGGTCGCGACCGAGGCGGAC<br>GGCGTCCCCTTCCACTTCCTCAATGGCCTTGACGTCGACCAAGCTACGGGTGATGCCT<br>ACTTCACCGACAGCAGCAGCACGTACACCCGCAGGTTCAACGGGGAGATCACGATG<br>AACGCCGACGCGACAGGGCGGCTGCTCAAGTACGACGCGCGGACGCGGCGGGTCA<br>CCGTGCTGAAGACCGACCTGCCGTACCCGAACGGCGTCGCCGTCAGCCGAGACAGG<br>ACGCACCTCGTCGTGGCGCACACCGTGCCGTGCCAGGCGTTCCGGTACTGGCTGCGG<br>GGCACCAAAGCCGGCGAGTACGAGCTCTTCGCCGACCTCCCGGGCTACCCCGACAAC<br>GAGAGGATGCGGCTGGGCGCGGCGCCCGCAGCCAAGCACCTGGTCGGCGTACGGC<br>TCAACCCCGACGGCGTGGAGGTGGAGGAGTTGACGGCTGCCAAGGGCGTCACGCTC<br>AGCGAGGTGGCGGAGCAGAAGGGCAAGCTGTGGTTGGGCTCCGTCGAACTCGATTA<br>CATTGGCATGTTTGCTTGA
**>STR2**	ATGGAAGAGAAGAAGCAGCAGCAGCAGCGTCCACAGAGAGGGCGCGATGGCATCC<br>TGCAGTATCCGCACCTTTTCTTCGCGGCGCTGGCGCTGGCCCTGCTCCTCACCGACCC<br>GTTCCACCTCGGCCCGCTCGCCGGGGTGGACTACCGGCCGGTGAGGCACGAGCTGG<br>CGCCGTACCGCGAGGTGATGGCGCGGTGGCCGCGGGACAACGGCAGCCGGCTCAG<br>GCACGGCAGGCTGGAGTTCGTCGGAGAGGTGTTCGGGCCGGAGTCCATCGAGTTCG<br>ACCGCCACGGCCGCGGCCCCTACGCCGGCCTCGCCGACGGCCGCGTCGTGCGGTGG<br>ATGGGGGAGGACGCCGGGTGGGAGACGTTCGCCGTCATGAGCCCTGACTGGTCGG<br>AGAAAGTTTGTGCCAATGGGGTGGAGTCGACGACGAAGAAGCAGCACGAGATGGA<br>GCGACGGTGCGGCCGGCCTCTCGGGCTGAGGTTTCACGGCGAGACCGGCGAGCTCT<br>ACGTCGCCGACGCGTACTACGGGCTCATGTCCGTCGGTCCGAACGGCGGGGTGGCG<br>ACCTCTCTCGCGAGAGAAGTCGGCGGGAGCCCGGTCAACTTCGCGAACGACCTCGAC<br>ATCCACCGCAACGGCTCCGTGTTCTTCACCGACACGAGCACGAGATACAACAGAAAG<br>GATCATCTGAACGTTCTGCTAGAAGGTGAAGGCACAGGGAGGCTGCTCAGATATGA<br>CCCAGAAACCAAAGCTGCCCATGTCGTGCTGAGCGGGCTGGTCTTCCCGAATGGCGT<br>GCAGATTTCTGACGACCAGCAGTTCCTCCTCTTCTCCGAAACAACAAACTGCAGGATA<br>ATGCGGTACTGGCTGGAAGGGCCAAGAGCCGGGCAGGTGGAGGTGTTCGCCGACC<br>TGCCGGGGTTCCCGGACAACGTGCGACTGAGCAGCGGCGGCGGCGGCGGACGGTT

	CTGGGTGGCGATCGACTGCTGCAGGACGGCGGCGCAGGAGGTGTTCGCCAAGCGG<br>CCGTGGCTGCGAACGCTCTACTTCAAGCTGCCCCTGACGATGCGGACGCTGGGGAA<br>GATGGTCAGCATGCGGATGCACACCCTCGTCGCGCTCCTCGACGGCGAAGGGGACG<br>TCGTCGAGGTGCTCGAGGACCGGGGCGGCGAGGTGATGCGGCTGGTGAGCGAGGT<br>GAGGGAGGTGGGGCGCAAGCTGTGGATCGGCACCGTGGCTCATAACCACATCGCCA<br>CGATCCCTTACCCGTTGGAAGAGCAGAGTAGCAGCAACGTGCTTGGTGATTGA
**>STR3**	ATGGTGCAGGGATGCCGATGCGAAGTAGTTGACGCCTGTGTTCGCGAGGTGCGCCT<br>CGCGTCGCCGTATCGTATCCATCAGATATTCAGATTCACAACCCCACGCCGCCTCGCC<br>CTCGCCCACCGCGGCGACACCTACAAAGCCCGCGACGAGGGCCAATGCCATTGGCGT<br>GACAAGGCGGGGAGCGCGACGCGACGCGAGGGCGGAGAGAGCGAGGGAGGGGA<br>GAGGAGGAGGAGGAGGTTGATGGCGTCGCCGGCGGTGGTGGCGTTCGCGGTGGCG<br>GTGGCGGTGGCGGCGCTGGCGGCGTTCTGCGGGACGGACCCGCTGCGGACGGGGA<br>GCATGGTGGACTTCCCGGGCTTCGTCCCCCACGTCGTCGAGCTCCCCGACGCGTCGG<br>AGATGCCGCCCCACGCGGACACCCGCGAGCGCCTCCGCGGCGCGGAGATCCGGTTC<br>CGCGGCGAGGTGCAGGGCCCCGAGAGCGTCGCGTTCGACCCGCTCGGCCGGGGAC<br>CCTACACCGGCGTCGCCGACGGCCGCGTCGTGCGCTGGGACGGCGCCCGCTGGGTC<br>TACTTCGCGCACTCCTCCCCGAACTGGACCGCCGAGCTCTGCGGCCACAAGGCGTCG<br>CCGCTCGACTACCTCAAGGACGAGCACATCTGCGGCCGCGCGCTCGGCCTCCGATTC<br>GACCGGAGGACGGGGGACCTCTACATCGCCGACGCCTACTTCGGCCTCCTCAAGGTC<br>GGCCCCGACGGCGGCCTCGCCACCCCGCTCGCCACCGAGGCCGAGGGCGTTCGCTTC<br>AACTTCACCAACGACCTCGACCTCGACGACGACGGCAACGTCTACTTCACTGACAGC<br>AGCATCCACTACCAGAGACGGCATTTCATGCAATTGGTTTTCTCTGGAGATCCCTCAG<br>GGAGGCTTCTGAAATACGACCCGAACACAAAGAAAGCAACAGTTCTCCACCGCAACA<br>TTCAGTTTCCGAATGGGGTGTCCATGAGCAAGGATGGCTTGTTCTTCGTCTTCTGCGA<br>AGGATCCCGTGGCAGATTGAGCCGATACTGGCTGAAAGGGGAGAAGGCTGGAACC<br>GTGGATCTTTTCGCCATCCTGCCTGGGTTTCCAGACAATGTGAGGACCAACGACAAG<br>GGTGAATTCTGGGTAGCAATCCATTGCAGGCGCAGCATATACGCCCGAATGGTCAGT<br>CGCAATGTCAGGCTGAGGAAGTTCTTGCTCAGCCTCCCAATCCCTGCCAAGTACCACT<br>ACCTGATGCAGATCGGCGGCAAGCTCCACGCTCTGATCATCAAGTACAACCCCGAAG<br>GCGAGGTGCTTGACATCTTGGAGGATACCACAGGGCAGGTGGTAAGAGCCGTTAGT<br>GAAGTTGAGGAGAAGGATGGGAAGCTCTGGATAGGATCCGTTCTGATGCCCTTCATT<br>GCCGTCTTCGATTACGCCAATGCATCTTAG
**>STR4**	ATGTGCGGGCGGCCGCTGGGGCTGCAGTTCCACCACGCCTCCGGCGACCTGTACGT<br>GGCCGACGAGTACCTGGGCCTCCTGAGGGCGCCGGCGCGCGGTGGGCTGGCCGAG<br>GTGGTGACGACGGAGACCGCCGGCGTGCCGTTCAACTTCCTCAACGGCCTCGATGTC<br>GACCAGAGGACCGGCGACGTCTACTTCACCGATAGCAGCAGCACGTACCGACAGCA<br>GCAGCACTTAATCGTGTGCCATTGCCACGCTCGCCCTCCGCCCGCTCCGCCGCCGCGC<br>TCGCCCTCCGCTTCTTTGCCCGTGGCCGCGCTCGCGCTCCACTTCTCGGCCCGCCGTC<br>ACTCCGCTGCCGCGCTCGCCCTCTGCTATGCCACCCGCATCCGCGCGCACCGCTCCGC<br>CTGCCGCCGGCCTCTTTCACTGAAGAAGAGGAATAGAGAAGAGAAGAGAAGGAGA<br>AGAAAAGAGAAGAAAAAAAATGTGCAGTTGTCATGTGGGTCCCATGTACTTTTTTTTT<br>AA
**>STR5**	ATGAGGACCACGGCGAGGCCAACGGCGGCGGCGACGGCGCTCGCGCTCATCCTCGT<br>CCTGGTGTTCTCGCCGTCCCCCGCCGCTGCCGCAGCGAGAATGTTCAAGACCATCGA<br>CGCCCGACGGAGCCAGCATCTGGACCTCGGCGGATCACTGGTCGGGCCAGAGAGCG

	TCGCGTTCGACGGCAAAGGCCGCGGCCCATACAGCGGCGTCTCCGACGGCCGCGTC<br>ATGAGGTGGAACGGCGAGGCGGCTGGCTGGAGCACCTACACGTACAGCCCCAGCTA<br>CACGAAAAACAAGTGCGCGGCATCGACTCTCCCCACGGTTCAAACCGAGAGCAAATG<br>CGGCCGTTCGTTGGGCCTACGGTTTCACTTCAAAACCGGCAACCTGTACATCGCCGAC<br>GCCTACATGGGATTGATGCGAGTTGGGCCAGGAGGTGGTGAGGCAACAGTGCTAGC<br>CACGAAGGCTGATGGCGTGCCACTTCGCTTCACCAATGGGGTGGACATTGATCAGGT<br>TACCGGAGATGTGTATTTCACTGACAGCAGCATGAACTACCAACGATCTCAGCACGA<br>GCAAGTCACGGCGACCAAGGATTCGACCGGACGGCTCATGAAGTATGACCCACGAA<br>CTAACCAAGTCACCGTTCTTCAATCCAACATAACCTACCCGAACGGTGTCGCCATTAG<br>CGCTGACCGAACACATTTGATCGTTGCGTTGACCGGCCCATGTAAGTTGAGGTATTG<br>GATTCGAGGTCCAAAGGTTGGTAAGTCCGAACCATTTGTCGACTTGCCAGGCTATCC<br>TGATAATGTGAGGCCTGATGAGAAAGGTGGTTACTGGGTAGCGCTCCATCGTGAGA<br>AGTATGAGCTTCCATTTGGTCCGGACAATCACTTGGTTGCAATGAGAGTTAGTGCTG<br>GTGGAAAGCTGGTGCAACAGATGAGAGGACCAAAGAGCTTGAGACCAACTGAAGT<br>GATGGAGAGGAAGGATGGCAAAATATACATGGGAAATGTTGAATTGCCATATGTCG<br>GAGTCGTCAAGAGTATCTAG
**>STR6**	ATGAGGACCACGGCGAGGCCAACGGCGGCGGCGACGGCGCTCGCGCTCATCGTCGT<br>CCTGGTGTTCTCGCCGTCCCCCGCCACCGCCGCCACAGCGAGAATGTTCAAGACCATT<br>GACGCCCGACGGAGCCAGCATCTGGACCTCACCGGATCACTCGTCGGGCCGGAGAG<br>CGTCGCGTTCGACGGCAAGGGACACGGCCCATACAGCGGCGTCTCCGACGGCCGCG<br>TCATGAGGTGGAACGGCGAGGCGGCTGGCTGGAGCACCTACACGTACAGCCCCAGC<br>TACACGAACAACAAGTGCGCGGCATCGACTCTCCCCACGGTTCAGACCGAGAGCAAA<br>TGCGGCCGTCCGTTAGGCCTCCGGTTTCACTTCAAAACCGGCAACCTGTACATCGCCG<br>ACGCCTACATGGGATTGATGCGAGTTGGGCCAGGAGGAGGGGAGGCAACCGTCTTA<br>GCCACGAAAGCTGATGGCGTGCCACTTCGCTTCACCAATGGGGTGGACATTGATCAG<br>GTTACCGGAGATGTTTATTTCACCGATAGCAGCATGAACTACCAACGATCTCAGCACG<br>AGCAAGTCACGGCGACCAAGGACTCGACGGGACGGCTTATGAAGTATGATCCACGA<br>ACCAACCAAGTCACCGTGCTTCAATCCAACATAACCTACCCGAACGGTGTCGCCATTG<br>GTGTTGACCGAACACATCTGATTGTTGCACTGACGGGGCCATGTAAGTTGATGAGGT<br>ATTGGATTCAAGGCTCAAAGGCTGGTAAGTCCGAACCATTTGCCGAGTTGCCAGGCT<br>ATCCTGATAATGTGAGGCCTGATGGGAAAGGTGGTTATTGGGTAGCCCTCCATCGTG<br>AGAAGTATGAGCTCCCCTTTGGTCCGGACAATCACTTGGTTGCTATGAGGGTTAGTG<br>CTGGTGGGAAGCTGGTGCAACAGATGAGAGGACCAAAGAGCCTGAGACCAACTGA<br>AGTGATGGGAGAGGAAGGATGGTAA
**>STR7**	ATGAGAAGCACGGCGAGGCAAGCGGCGACCGCGGCGGCGTTCGCGCTCATTGTCTT<br>CCTCGTGCTGCTCTCGCCGTCCCCTACTGCCGCCGCCACAGCCACAACGAGAATGTTC<br>AAGACCATTGACGCCCGGCGGAGCCAGCATCTGGACCTCGGCGGATCACTGGTCGG<br>CCCGGAGAGCGTCGCGTTCGACGGCAAAGGCCGCGGCCCGTACAGCGGCGTCTCCG<br>ACGGCCGCATCATGAGGTGGAACGGCGAGGCCGCTGGCTGGAGCACCTACACGTAC<br>AGCCCCAGCTACACGAAAAACAAGTGCGCGGCATCGACTCTCCCCACGGTCCAGACC<br>GAGAGCAAATGCGGCCGCCCGTTAGGCCTACGGTTTCACTACAAAACCGGCAACCTG<br>TACATCGCCGACGCCTACATGGGATTGATGCGAGTTGGTCCAAAAGGCGGGGAGGC<br>AACCGTGCTAGCCATGAAGGCTGATGGCGTGCCACTTCGCTTCACCAATGGGGTGGA<br>CATTGATCAGGTTACCGGAGATGTTTATTTCACCGACAGCAGCATGAACTACCAACG<br>ATCTCAGCACGAGCAAGTCACGGCGACCAAGGATTCGACCGGACGGCTCATGAAGT<br>ATGACCCACGAACTAACCAAGTCACCGTTCTTCAATCCAACATAACCTACCCGAACGG

	TGTCGCCATGAGCGCTGACCGAACACATCTGATCGTTGCATTGACCGGGCCATGTAA GTTGATGAGGCATTGGATCCGAGGCCCGAAGACTGGCAAATCTGAACCATTTGTTGA CCTGCCAGGCTATCCTGATAATGTGAGGCCTGATGGAAAAGGTGGTTATTGGATAGC GCTTCATCGCGAGAAGTATGAGCTTCCCTTTGGTCCGGATAGTCACTTGGTTGCTATG AGGGTTAGTGCTGGTGGGAAGCTGGTTCAACAGATGAGAGGACCAAAGAGCTTGAG GCCAACCGAAGTGATGGAGAGGAAGGATGGCAAAATATACATGGGAAATGTTGAAT TGCCGTATGTCGGAGTCGTCAAAAGCAGCTAG
**>STR8**	ATGGGGGCCGTCCTCGGCACCGGGAGGGTGGGGACTCTGACTCGGGTGGCGCTGA CGATCGTCGTCTTCCTGCTGCTGTTGCCGTCGCACGCCCTCGCCGCGGCCGTCGCGAA GGACACCTCCGCCACACTGGTCGAGACGCTGCCGCTGCCCACGACGCTGGTCGGCCC AGAGAGCGTCGCGTTCGACAAGTTCGGCGATGGCCCCTACAGCGGCGTCTCCGACG GCCGCATCCTCCGCTGGGACGGCGCCGACAAAGGCTGGACGACGTACTCCCACGCCC CGGGGTACAACGTCGCCAAGTGCATGGCTCCCAAGCTCCATCCCGCGGAGCTCACCG AGAGCAAGTGCGGCCGGCCGCTCGGCCTCCGGTTCCACAACACCTCCGGTAACCTCT ACATCGCCGACGCGTACAAGGGCCTCATGCGTGTCGGCCCGCGCGGCGGGGAGGCA ACGGTGCTCGCCACGGAGGCCGACGGCGTGCCGTTCAAGTTCACCAACGGCGTCGA CGTCAACCAGGTCACCGGCGAGGTCTACTTCACCGACAGCAGCACGCGCTTCCAGCG ATCCCAGCACGAGATGGTCACGGCCACCGGCGACTCCACGGGCCGCCTGATGAAGT ACGACCCGACGACGGGCTACCTCGACGTGCTCCAGTCCGGAATGACGTACCCGAACG GCCTCGCCATTAGCGCCGATCGGAGTCACCTCGTGGTGGCGCTGACGGGGCCGTGC AAGCTGGTGAGGCACTGGATCGAGGGCCCCAAGGCCGGTACGTCGGAGCCGTTCGC CGAGCTGCCGGGCTACCCGGACAACGTGAGGCCCGATGGGAAGGGAGGCTACTGG GTGGCGCTGCACCGCGAGAAGACCGAGACGCCGTATGGCTCGGACACCCACCTCCTC GCCGTAAGGATCGGTCGCAAGGGGAAGATCTTGCAGGAGTTGAGGGGGCCGAAGA ACGTCAGGCCAACGGAGGTGATTGAGAGAGGCGGCGGCAAGCTTTATTTGGGTTCG GTGGAATTGGGTCATGTGGCTGTTGTTAAAGCTACTTAG
**>STR9**	ATGGCTCCCAAGCTCCATCCCGCGGAGCTCACCGAGAGCAAGTGCGGCCGGCCGCTC GGCCTCCGGTTCCACAACACCTCCGGTAACCTCTACATCGCCGACGCGTACAAGGGC CTCATGCGTGTCGGCCCGCGCGGCGGGGAGGCAACGGTGCTCGCCACGGAGGCCG ACGGCGTGCCGTTCAAGTTCACCAACGGTGTCGACGTCAACCAGGTCACCGGCGAG GTTTACTTCACAGACAGCAGCACGCGCTTCCAGCGATCCCAGCACGAGAGGGTCACG GCCACCGGCGACTCCACGGGCCGCCTGATGAAGTACGACCCGACGACGGGCTACCT CGACGTGCTCCAGTCCGGAATGACGTACCCCAACGGTCTCGCACTCAGCGCCGATCG GAGTCACCTCGTGGTGGCGCTGACGGGGCCGTGCAAGCTGGTGAGGCACTGGATCG AGGGCCCCAAGGCCGGTACGTCGGAGCCGTTCGCCGAGCTGCCGGGCTACCCGGAC AACGTGAGGCCCGATGGGAAGGGAGGCTACTGGGTGGCGCTGCACCGCGAGAAGA CCGAGACGCCGTATGGCTCGGACACCCACCTCCTCGCCGTAAGGATCGGTCGCAAGG GGAAGATCTTGCAGGAGTTGAGGGGGCCGAAGAACGTCTGGCCAACGGAGGTGAT TGAGAGAGGCGGCGGCAAGCTTTACTTGGGTTCAGTTGAATTGGGTCATGTCGCCGT CGTCAAGGCTAGCGCTACTTGA
**>STR10**	ATGGCTCCCAAGCTCCATCCCGCGGAGCTCACCGAGAGCAAGTGCGGCCGGCCGCTC GGCCTCCGGTTCCACAACACCTCCGGTAACTTCTACATCGCCGACGCGTACAGGGGC CTCATGCGTGTCGGCCCGCGCGGCGGGGAGGCAACGGTGCTCGCCACGGAGGCCG ACGGCGTGCCGTTCAAGTTCACCAACGGTGTCGACGTCAACCAGGTCACCGGCGAG GTCTACTTCACCGACAGCAGCACGCGCTTCCAGCGATCCCAGCACGAGAGGGTCACT

	GCCACCGGCGACTCCACGGGCCGCCTGATGAAGTACGATCCGACGACGGGCTACCTC<br>GACGTGCTCCAGTCCGGAATGACGTACCCCAACGGTCTCGCGCTCAGCGCCGATCGG<br>AGTCACCTCGTGGTGGCGCTGACGGGGCCATGCAAGCTGGTGAGGCACTGGATCGA<br>GGGCCCCAAGGCCGGTACGTCGGAGCCGTTCGCCGAGCTGCCGGGCTACCCGGACA<br>ACGTGAGGCCCGATGGGAAGGGAGGCTACTGGGTGGCGCTGCACCGCGAGAAGAC<br>CGAGACGCCGTATGGCTCGGACACCCACCTCCTCGCCGTAAGGATCGGTCGCAAGG<br>GGAAGATCTTGCAGGAGTTGAGGGGGCCGAAGAACGTCAGGCCAACGGAGGTGAT<br>TGAGAGAAGCGGCGGCAAGCTTTACCTGGGTTCAGTTGAATTAGGTCATGTCGCCGT<br>TGTTAAGGCTACTTGA
**>STR11**	ATGGGCGCCGTCCTCGGCACCGGGAGGGTGGGGACTCTGACTCGGGTGGCGCTGAC<br>GATCGTCGTCTTCCTGCTGCTCCTGCCATCGCACGCCCTCGCCGCGGCCGTCGCGAAG<br>GACACCTCCGCCACACTGGTCGAGACGCTGCCGCTGCCCACGACGCTGGTCGGCCCG<br>GAGAGCGTCGCGTTCGACAAGTTCGGCGATGGCCCCTACAGCGGCGTCTCCGACGG<br>CCGCATCCTCCGTTGGGACGGCGCCGACGAAGGTTGGACGACGTACTCCCACTCCCC<br>GGGGTACAACGTCGCCAAGTGCATGGCTCCCAAGCTCCATCCCGCCGAGCTCACCGA<br>GAGCAAGTGCGGCCGGCCGCTCGGCCTCCGGTTCCACAACACCTCCGGTAACCTCTA<br>CATCGCCGACGCGTACAAGGGCCTCATGCGTGTCGGCCCGCGCGGCGGGGAGGCAA<br>CGGTGCTCGCCACGGAGGCCGACGGCGTGCCGTTCAAGTTCACCAATGGTGTCGAC<br>GTCAACCAGGTCACCGGCGAGGTCTACTTCACCGACAGCAGCACGCGCTTCCAGCGA<br>TCCCAGCACGAGATGGTCACGGCCACCGGCGACTCCACGGGCCGCCTGATGAAGTA<br>CGACGCGACGACGGGCTACCTCGACGTGCTCCAGTCCGGAATGACGTACCCCAACG<br>GCCTCGCCCTTAGCGCCGATCGGAGTCACCTCGTGGTGGCGCTTACGGGGCCGTGCA<br>AGCTGGTGAGGCACTGGATCGATGGCCCCAAGGCCGGTACGTCGGAGCCATTCGCC<br>GAGCTGCCGGGCTACCCGGACAACGTGAGGCCCGACGGGAAGGGAGGCTACTGGG<br>TGGCGCTGCACCGTGAGAAGACGGAGTCGCCGTACGGCTCGGACACCCACCTCCTC<br>GCCGTGAGGATCGGTCGCAAGGGGAAGATCTTGCAGGAGTTGAGGGGGCCGAAGA<br>ACGTCAGGCCAACGGAGGTGATTGAGAGAGGCGGCGGCAAGCTTTACTTGGGTTCA<br>GTTGTGTGGAGATTATGGGTACCCCATACCCACACGGCATAG
**>STR12**	ATGGCGCAGCAGCTGGGCCTCCTCGCCGCCGTCTTCGTGTCGCTCGCCGTGCACGTC<br>GCGCTCCACTGCCCCATCCAGCCGCTCGCCCCGCCGCCCGCACGCCCACCCGCCGCC<br>GCTCGCTTCCCTCCCAACAACCTCCTCCAGAATCTGGAGAAGCTTGGGGAAGGGATG<br>CTGAGCGCGCCGGAGGACGTGTACGTGGACGACGCCGGCGGCGAGGTGTTCACGG<br>CGACGAGGGACGGGTGGGTGCGGAGGATGCAGGCGAACGGGTCGTGGGAGCGGT<br>GGGGGCTCGTCGGCGGCACGGGGCTCCTCGGCGTCGCCCCGTCCGCCGACGGCGCC<br>ATGCTCGTCTGCGACGCCGACAAGGGATTGTTGAAAGTGGATGAGAATGGACGCGT<br>GACGCTTCTTGCTTCGACTGTCGAAGGCTCCACGATCAGGTTCGCGGACGCGGCGAT<br>CGAGGCCTCCGATGGCACGGTGTACTTCAGCGACGCCAGCACCAGGTTCAGCTTCGA<br>CAACTGGTTCCTCGACTTCTTCGAGTACCGCTTCACCGGCCGCCTGCTCAAGTACGAC<br>CCCCGCACCGGCGAGGCCTCCGTCGTGCTCGACGGCCTCGGCTTCGCCAACGGCGTC<br>GCCCTGCCGCCCGACGAGGCCTTCGTCGTCGTCTGCGAGACGATGAGGTTCAGATGC<br>TTGAGAGTGTGGCTGAAAGGGGAGAAGGCTGGGGAGGCAGAGATCTTCGTGGACA<br>ACCTGCCGGGGAATCCAGACAACATTCGGCTGGGTTCAGATGGTCACTTCTGGATTG<br>CCCTTCTCCAGGTGAGGTCTCCATGGCTGGACCTGATCTCTCGCTGGAGCTTGACGA<br>GGAGGGTCATCGCGTCGTTCCCGGCGCTCGTCGAGAGGACCAAGGCGACGCTCAAG<br>GGAGCAGTGGTGGCTCAGGTGTCGTTGAACGGCGAGATCGTGAGGGTTCTTGGTGA<br>CTCTGAAGGGAATGTGATCAACATGGTCACTTCGGTGACAGAGTTCAACGGAGATCT

	CTTCCTCGGCAGCCTTGCGACCAACTTCATAGGAAAATTATCCCTGGCTAAGGTTACA CGGGAGCAGGAGGATGCAGTTCCTTCGTAG
**>STR13**	ATGGCTCGCCGTCGTCGAGGTTTTCCGGCGACGTTGGTCACGCTGCTGCGGCTCGTC GGCTGCCTGCTTCTTGCCTTCTTCCTCGCCGCGCCGCCGTGCGCCGCCGCGCAGCAGG TCAAGACGTCGCACGCGCAGTTCGCGTTCCACCTCCCGCTCCCCGACGGCGTCACCG GCGCCGAGAGCCTCGCCTTCGACTCCTCCAACCATGGCCCCTACACCGGCGTCTCCGA CGGCCGCGTCCTCCGCTGGGGCGGCGCCGCCGCCGGCTGGACCACCTTCGCGCACC ACGAAAACTACCGGAAGATTCCGATGTGCACGACGCCGGTGGCGCCGGCGGAGGA GACGGAGAGCATGTGCGGGCGCCCGCTGGGGCTCGCGTTCCACGACAGGACGGGC GACCTCTACATCGCCGACGCGTACAAGGGCCTGATGCGTGTCGGGCCGCGCGGCGG CGAGGCCGAGGTGCTCGCCGCCGGCGCGGACGGCGTCCCGTTCAACTTCGTCAATG GCATCGACGTCGACCAGGCCACCGGTGATGTCTACTTCACCGATAGTAGCACCACCT ACCCACGCCGGTTTAACAGCGAGATCATGATGAATGCCGATGCGACGGCGCGGCTG CTAAAGTACGACGCGGCGACGAAGCGGGTGACCGTGCTCAGGGCCGGGCTTCCGTA CGCCAATGGCGTGGCCGTCAGCCGCGACGGGAGCCACGCCGTGGTGGCGCACACG GTGCCGTGCCAGGCGTTCCGGTACTGGATCAAGGGGCCCAACGCCGGCGAGTACGA GCTCCTCGCCGACCTGCCGGGTTACCCCGACAACGTCCGGCGAGACGCCAATGGGG GATACTGGGTGGCGCTCAACCAGGAGAAGGCGCGGCTCGACGCCACCGCGGCGGC GGCGGTGGCTCCTCCGGCGAAGCACCTCGTCGGCGTCCGGCTCGACGGCGATGGCG TCGAGGTCGAGGAGCTGACGGCTGCCAAGGGTGTGACGCTCAGTGAGGTTGTGGA GAGGGGCGGGAAATTGTGGCTCGGCTCTGTTGAACTCGATTTCATTGGCCTAATGCA ATGA
**>STR14**	ATGGCTAGTTTCTCGACGGTTTTCCTGCTCCTCAGCCTTGTCTGCCTAGCTTCGCCGTG CGCCGCGCAGCGCGTGATCTGCGAAGCCCCACCGTGCACCACCGCGTCGCAGGTGA AGACAACCCCGACAAATCGCGCCTCCCACGTCCGCCTCCCCGAAGGCGTCACCGGCG CCGAGAGCCTCGCCTTCGACTCCAGCAACCGTGGCCCCTTCACCGGCGTCTCCGATG GCCGCGTCCTCAAGTGGGGCGGCGACTCCGCCGGCTGGACTACCTTCGCGTACAACC GGAATTACCGGAGCAACCCTACGTGCGCGTCGTCATCTGAGGAGACGGAGAGCACA TGCGGGCGTCCGCTGGGTCTTGCGTTCCACCTCAAGACGGGGATCCTCTACTTCGCC GACGCCTACAAGGGCCTGATGCGGGTTGGGCCGCGGGGTGGCCAGGCCGACGTGC TCGCCACGGAGGCCGACGGCGTGCCGTTCAATTACCTCAACGGCGTCGACGTCGACC AGGACACCGGTGACGTCTACTTCACCGACAGCAGCACCACCATCACACGCCGATACC AAGAGAACATCATGAGGAACCGCGACGCGACGGCTCGGCTGATGAAGTACGACGCG AAGACGAAGCAGGTGACGGTGCTGAAGGACCGGTTGCCGTACGCCAACGGCGTGG CCGTCAGCCACGACGGGAGGTACCTCGTGGTGGCGCACACGGGGCCGGCACAGGT GTTCAGGTATTGGCTCAAGGGGGCCAAGGCCGGCCAGTACGAGCTCTTCGCCGACCT GCCCGGGTACCCCGACAACGTCCGGCGAGACGCCAAGGGGGGCTACTGGGTGGGG CTCAACCGGGAGAAGATTACGTTCAACGTGCCGGCGGCGGCTTCTCCGGCCAAGCAC CTGGTCGGCGTCCGGCTCAACGGCGACGGCGTCGAGGTGGAGGAGCTGACGGCTG CTAGTAGGGCTGTGACGCTGAGCGAGGTTGTGGAGAGGGACCGCAAGTTGTGGCTC GGCTCCGTCGATCTCGACTACGTCGGCCTACTGCAGTAG
**>STR15**	ATGAGGAAAGGCGCCGCCGGAATGGCGTGCACTTGCTCGGCGGCGGCGGCGGCCT CCGCTCTTGTGAAGCTTCTTGTTCTTGTGGCGGCGGTGGCGGCGACGACGTCCGCCG GTGGCGGCGACGAGCCGACGTACGAGACCAAGTCCATAGACCCGAGCCTCGCCGTG ATGACGCTGCCGGCGCCCGTGACGGGCCCGGAGAGCCTCGCCTTCGACGGCCGCGG CGACGGGCCCTACACCGGCGGCTCCGACGGCCGCATCCTCCGCTGGCGCGGCGGCC GCCTCGGCTGGACCGAGTTCGCCTACAACTCCAGGCACAAGAGCGTCGGCGTTTGCT

	CGCCGGAGAAGAAGCTGGTGGTGCCGGAGAGCGTGTGCGGGCGGCCGCTGGGGCT<br>GCAGTTCCACCACGCCTCCGGCGACCTATACGTCGCCGACGCGTACCTGGGCCTCCT<br>GAGGGTGCCGGCGCGCGGCGGGCTGGCCGAGGTGGTGGCGACGGAGGCCGCCGG<br>TGTGCCGTTCAACTTCCTCAACGGCCTCGACGTCGACCAGAGGACCGGCGACGTCTA<br>CTTCACCGATAGCAGCACCACGTACCGGAGGAGCCAGTACCTGCTGGTGGTGGCCAT<br>GGGTGACGAGACGGGGCGGCTGCTCCGGTACGACGCGCGGCGGCGGCGCGTCACC<br>GTGCTCCACTCCGGCCTCCCCTACCCGAACGGCGTCGCCGTCAGCGACGATGGCACC<br>CACGTGGTGGTGGCCCACACGGGCCTCTGCGAGCTCCGCCGCTACTGGCTGCGCGG<br>GCCGCGCGCCGGCAAGTCGGAGACGTTCGCCGAGGTGCCCGGCTACCCGGACAACG<br>TGCGCCGCGACGGCGACGGCGGGTACTGGGTGGCGCTCAGCCGCGGGGCCGACAA<br>TGACGACGTAGCGCCGACCGTGGCCGTGCGGGTGACGGCGGCGGGGAAGAAGAAG<br>GGTGGAGGCGCGGCGGTGGTGGCAGAGGCGCTCGCCGGGTTCAGCTTCGTGACGG<br>TGAGCGAGGTGGCGGAGCAGAATGGCACGCTCTGGATCGGCTCCGTCGACACGCCG<br>TACGCCGGCGCCGCGGTGAGGGGTCGCCGGTGA
**>STR16**	ATGAGGAAAGCCGGCAGAAGGGCGTTCACTTGCTCGGCGGCGGCGGCGGCGG<br>CGGCCTCCGCTCTTGTGAAGCTTCTTGTTCTTGTGGCGGCGGTGGCGGCGACGACGT<br>CCGCCGGTGGCGGCGACGAGCCGACGTACGAGACCAAGTCCATAGACCCGAGCCTC<br>GCCGTGATGACGCTGCCGGCGCCCGTGACGGGCCCGGAGAGCCTCGCCTTCGACGG<br>CCGCGGCGACGGGCCCTACACCGGCGGCTCCGACGGCCGCATCCTCCGCTGGCGCG<br>GCGGCCGCCTCGGCTGGACCGAGTTTGCCTACAACTCCAGGCACAAGAGCATCAGCG<br>TTTGCTCGCCGGAGAAGAAGCTGGTGGTGCCGGAGAGCGTGTGCGGCCGGCCACTG<br>GGGCTGCAGTTCCACCACGCCTCCGGCGACCTGTACGTGGCCGACGCGTACCTGGGC<br>CTCCTGAGGGCGCCGGCGCACGGCGGGCTCGCCGAGGTGGTGGCGACGGAGGCCG<br>CCGGCGTGCCGTTCAACTTCCTCAACGGCCTCGACGTCGACCAGAGGACCGGCGACG<br>TCTACTTCACCGATAGCAGCACCACGTATCGGAGGAGGGTGAAAAATTTTGAAGTGG<br>AATCTAAACACCCCCTAACCCTACATCACGAAGTTATCCGTAAATATCCCTACAAATAT<br>CCATAG
**>STR17**	ATGGGCGACGAGACAGGGCGGCTGCTCTGGTACGACGCGCGGCGGCGTCACGTGA<br>CCGTGCTCCACGCCGGCCTCCCGTACCCGAACGGCGTCGCCGTTAGCGACGACGGCA<br>GCCACGTGGTGGTGGCCCACTCGGGCCTCTGCGAACTCCGCCGCTGCTGGCTGTGCG<br>GGCCCAGCGCTGGCAAGTCAGAGACGTTCGCCGAGGTGCCCGGCTACCCGGACAAC<br>GTCCGCCGCGATGACAGCCGCGGCGGGTACTGGGTGGCGCTCAGCCGCGAGGCCG<br>ACAGTGATGACATGGCGCCGACGGTGGCTGTGAGGGTGGTGGCGCCGGCCGCGAA<br>GAATGGTAGCGCGGCGGTGGTGGCGGAGGCGCTCGCCGGATTCAGCTTCGTGACG<br>GTGAGCGAGGTGGCGGAGCGGAACAGCACGCTTTGGGTCGGTTCCGTTGACACGCC<br>GTACGCCGGCGCTGCGGTGAGGGGTCACCGGTGA
**>STR18**	ATGTGGCCGTGCTTGTCTCCCCGGGCGGCAGTGCTCATTGCGCTCCTCCTCTTGCCGG<br>CGGCCATGGCGGCGGCGGCGGCGGCGGCCGGCGGCCACGGCGAGGAGATGAAGT<br>CCATCTACGCCGGGCCCAAGGTCGTGCCCGTGCGGCTGGGCCGGCCGGCGTTCGGC<br>CCGGAGAGCCTCGCCTTCGACCACCGCGGCGGCGGCCCCTACACCGGCGTCTCCAAC<br>GGCCGCGTCCTCCGGTGGCGCGCCGACCGCCGCCGCCCCGGCTGGACCGAGTTCGC<br>CCACAACTACAAGCACGCGACGGTGGCGGAGTGCGCAGCGAGGAAGAAGGCGGCG<br>GCGGCGGCGGAGAGCGTGTGCGGGCGGCCGCTGGGGGTGCAGTTCGACCGGAGG<br>ACGGGCGAGATGTACATCGCGGACGCGTACCTGGGGCTGATGAGGGTGGGGCGGC<br>GCGGCGGGATGGCGGAGGTGGTGGCGGCGGAGGCCGGCGGCGTGGCGCTCAACTT

	CGCGAACGGGGTGGACGTCGACCAAGCCACCGGCGACGTCTACTTCACCGATAGCA<br>GCACCACGTACAAGCGGAGCGACTACCTGCTGGTGGTGCTCTCCGGCGACGCGACG<br>GGGCGGCTGCTCCGGTACGAGCCGCGGACGGGCAACGTCACCGTGCTCGAGTCCGG<br>CCTCGCCTTCCCCAACGGCGTCGCCGTCAGCGCCGACGGCACCCACCTCGTCGTCGCC<br>GAGACGGCCTCCTGCCGGCTGCTCCGCCACTGGCTCCGCGGCAGCAACGCCGGCGC<br>CACCGAGGTTCTCGCCGACCTCCCCGGCTACCCGGACAACGTGCGCCACGCCGCCGC<br>CGACGGCGGCCGCGGCGCGTCGTACTGGGTCGCGCTCAACCGCGACAAGGCGTGGA<br>CCGTGAACGGCACGACGCCGGCGTCCGTGGCCGCCGTCAGGGTGGTCGTCGACGAC<br>GGCGGCAGCAAGGTGGACGTGGCGCTGCGCGGGTTCGGCGGCGCCACGGTGAGCG<br>AGGTGGTGGAGCGGAACGGGTCGCTGTGGTTCGGCTCCGTCGACACGCCGTACGTC<br>GGCTTGCTCAAGCTCACCTCGCTCTAG
**>STR19**	ATGGCGTCGTCGTTGTCGTTGCTGCTCGCCGGCGTGAAGGCGGCGCTGGTGGTGCTC<br>GCCGGCGTCGCGCTGTACAGCCCCGAGGGGTTCTCGCCGGCGCCGATGCCGCCCGA<br>GTACTCGTACGGCGCCCCCGTGTCGGCGCCGCGGCACGAGCCCCGCGCGCTGGCGG<br>CGAGCGAGCGCGTCGGGGAGGGGCGGCTCCCGGCGCCGGAGGACCTGGCCTACGA<br>CGCCGCCGGCGGGTGGCTGTACACCGGCTGCGGCGACGGGTGGGTTCGCAGGGTG<br>AGCGTCTCGTCCGGGGACGTCGAGGACTGGGCGCGCACCGGCGGCCGCCCCCTCGG<br>CGTCGCCCTCACCGCCGACGGCGGCCTTGTCGTCGCCGACGCCGACATCGGGTTACT<br>GAAGGTGAGCCCGGACAAGGCGGTGGAGCTGCTGACCGACGAGGCGGAGGGCGTC<br>AAGTTCGCCCTGACCGACGGCGTCGACGTCGCCGGCGACGGCGTCATCTACTTCACC<br>GACGCGTCGCACAAGCACAGCCTCGCGGAGTTCATGGTGGACGTGCTCGAGGCGCG<br>CCCCCACGGGCGGCTGATGAGCTTCGACCCGTCGACGCGGCGGACCACCGTGCTCG<br>CCCGCGGCCTCTACTTCGCCAACGGCGTCGCCGTCTCGCCGGACCAGGACTCCCTCGT<br>CTTCTGCGAGACCGTCATGAGGAGGTGCTCGAGATACCACATCAACGGCGACAAGG<br>CCGGCACCGTCGACAAGTTCATCGGCGACCTGCCGGGCTTCCCTGACAACATCCGCT<br>ACGACGGCGAGGGCCGCTACTGGATCGCCATCTCCGCCGGGAGGACGCTGCAGTGG<br>GACGTGCTGACGAGGTCGCCGTTCGTGAGGAAGCTGGTGTACATGGTGGACAGGTT<br>CGTCGTGGCGGTGCCCCACAACCTGAAGAACGCCGGCGCCATGAGCGTGACGCTCG<br>CCGGAGAGCCCGTGTCGATGTACAGCGACCCGGGACTCGCCCTCACCACCGGCTGG<br>CTCAAGGTCGGCGACTACCTCTACTACGGCTCGCTGACCAAACCGTACCTCAGCAGG<br>ATCGACCTCGCCAAATCGCCAGCTGAGAAGGCTCAGGAGTGA
**>STR20**	ATGAACACCACGGCCAAGCTCTTGGCGCTCGCCGTCTTCGCGGCGGCGGCGATCTTG<br>TCGCTGGACTCGCGGAGCGACGTGAGGCAGCTGGAGATAAGGGACGGCGACGTCG<br>AGCTGATCCCTCTGCTCGACGGCGCCGCGGGGCCGGAGAGCATAGTGTTCGGCGAC<br>GCCGGCGATGGGCCGTACACGAGCGTGTCTGACGGGAGGATCCTCAAGTGGCTGCC<br>GCCGCCGGAGCGCCGGTGGGTCGAGCACTCCTGCTCCGTGCCGGAGCTGTTGGATA<br>GCTGCAGAGGATCCAAGGACACGAAACGGGAGCAGGAGTGTGGGCGTCCACTGGG<br>CCTCAAGTTCAATAGCAAGACTGGTGAGCTGTACGTCGCAGATGCGTACCTTGGGCT<br>GAGAGTGGTCAGTCCGGGTGAGAACGTGTCTAGGCCGCTTGTTCCTAAGAGGACAG<br>GAAGCCCATTCAGCTTCTCCAACGGCGTTGAGATTGACCATGAAACTGGAGTAATCT<br>ACTTCACCGAGACCAGTACAAGGTTTCAGAGAAGGGAGTTTCTAAACATAGTTATAA<br>CGGGTGACAACACTGGAAGATTGTTGAAATACGATCCAAAAGAAAACAAGGTTGAA<br>GTCTTAGTTGATGGCCTACGTTTTCCTAATGGTTTGGCTATGAGCATCGATGGTTCTT<br>ATTTGCTACTTGCGGAAACCACAACGGGTAAGATCCTAAGATATTGGATTAAAACAC<br>CAAAAGCATCAACTATTGAAGAAGTTGCGCAACTACCTGGGTTTCCAGACAACATTA<br>AGATGAGTCCTAGAGGAGGGTTTTGGGTTGGTCTTCATGCCAAGAGAGGGAAGATC

	GCTGAGTGGTCAATTTCTTATCCTTGGCTAAGGAAACTAATCTTTAAGCTACCAGCTC<br>AACGCATTCAACGCATCACATCGTTCTTGACAGGATTTGGTCGTCAAGTGATAGCTTT<br>GAGGTTGAGTGAGGATGGGAAGACCATAGAAGCAATGAGTGTTCATGGTGATGTCA<br>GGAAGTTGTTCAAGTCTATTAGCGAAGTTGAAGAAAAGGATGGGAACCTCTGGATA<br>GGATCTGTTTTGTCACCTTTTCTTGGGCTTTATCGTATATAG
**>STR21**	ATGATTTGGTGGAATATCATGTCAAGGAGCAAATGCGACAAGGCAATCAACCATATC<br>TCTACTCTACATAAACAAATCCGGAGCCGTTCACGCGTTGCAATTTCATTAATCGCAG<br>TTACAATTTCATTAATCGCAGTTATTCACATAGGTTCTGGACTTCTGGTTCCGATGAAA<br>GCCAGGCTCGTGGTGCTCGCGGCCGCCGTGGCGGCGGCGGCGTTGTTGGTGTCCCT<br>GGACCCCCGGAGCGACGACGTGCCAGTGCTGGAGATATGGGAGCGCGACGTCGAG<br>CTTATCACCGTGGACGCCGGCGGCGCGGTCGGGCCGGAGAGCGTGGCGTTCGACGG<br>CGACGGCGACGGCCCGTACACGGGCGTGTCGGACGGGAGGGTGCTCAAGTGGCTTC<br>CCCTGGAGCGCCGCTGGGTCGAACACTCGTCCGCCGTCATCGAGCCACATATGTTGG<br>ATAGCTGCAGAGGATCCAAGGACACGAAACGGGAGCAGGAGTGTGGGCGTCCACT<br>GGGCCTCAAGTTCAATAGCAAGACTGGTGAGCTGTACGTCGCAGATGCGTACCTTGG<br>GCTGAGAGTGGTCAGCCCGGGTGAGAACGTGTCTAGGCCGCTTGTTCCTAAGTGGA<br>CAGAAAGCCCATTCAGCTTCTCCAATGGCGTTGAGATTGACCATGAAACTGGAGTAA<br>TCTACTTCACCGAGACCAGTACAAGGTTTCAGAGAAGGGAGTTTCTAAACATAGTTAT<br>AACGGGTGACAACACTGGAAGATTATTGAAATATGATCCAAAGGAAAACAAGGTTG<br>AAGTCTTAGTTGATGGCCTATGTTTTCCTAATGGTTTGGCTATGAGCAACGATGGTTC<br>TTATTTGCTACTTGCGGAAACCACAACGGGTAAGATCCTAAGATATTGGATTAAAACA<br>CCAAAAGCATCAACTATTGAAGAAGTTGTGCAACTACATGGGTTTCCAGACAACATC<br>AAGATGAGTCCTAGAGGAGGGTTTTGGGTTGGTCTTCATGCCAAGAGAGGGAAGAT<br>CGCTGAGTGGTCAATTTCTTACCCTTGGCTAAGGAAAGTAATCTTGAAGCTACCAGCT<br>CAACGCATTCAACGCATCACATCGTTCTTGACAGGATTTGGTCGTCAGGTGATAGCTT<br>TGAGGTTGAGTGAGGATGGGAAGACCATAGAAGCAATGAGTGTTCATGGTGATGTT<br>AGGAAGTTGTTCAAGTCTATTAGCGAAGTTGAAGAAAAGGATGGGAACCTCTGGAT<br>AGGATCTGTTTTGTCACCTTTTCTTGGGCTTTATCGTATATAG

Supplementary method

Method S1. Chromosomal distribution of *ZmSTRL* genes

The chromosomal positions of the identified ZmSTRL genes were retrieved from Maize-GDB (https://maizegdb.org) according to the B73_v4 maize reference genome annotation. The physical distribution of ZmSTRL genes on maize chromosomes was subsequently visualized using MG2C (http://mg2c.iask.in/mg2c_v2.1/). The chromosomal distribution pattern was further used to evaluate possible local clustering of homologous ZmSTRL genes and to support cautious interpretation of potential duplication related contributions to family diversification.

Method S2. Cis-regulatory element analysis of *ZmSTRL* promoters

To explore the potential regulatory characteristics of *ZmSTRL* genes, 2000-bp upstream sequences of each gene were extracted from the B73_v4 maize reference genome and considered as promoter's region. These promoters sequences were analyzed using PlantCARE (https://bioinformatics.psb.ugent.be/webtools/plantcare/html/) for the identification of cis-acting regulatory elements [1]. The identified regulatory elements and their distribution patterns were subsequently illustrated using GSDS.

Method S3. Phylogenetic analysis, gene structure and conserved motif characterization

The complete STR proteins sequences were initially aligned using CLUSTALW with default alignment parameters. To investigate the evolutionary relationships of maize STRL genes within a broader plant context, STRL protein sequences from maize, rice (*O. sativa*) and *A. thaliana* were included in the comparative phylogenetic analysis. Poorly aligned terminal regions and obvious alignment gaps were checked manually before tree construction. The aligned sequences were subsequently used for phylogenetic reconstruction in MEGA 6.0. The phylogenetic tree was generated using the neighbor-joining method and the reliability of individual branches was assessed using 1000-bootstraps replicates. The neighbor joining approach was used to classify STRL proteins into major evolutionary groups and compare subgroup distribution among maize, rice and Arabidopsis. The resulting tree was visualized and edited using EvolView (https://www.evolgenius.info/evolview/) [2].
Gene structure analysis of *ZmSTRL* members was performed using annotation information from the B73_v4 maize reference genome. Exon–intron organization were visualized using the Gene Structures Display Server (GSDS). Conserved protein domains were identified by searching STR proteins sequences against the Pfam databases (http://pfam.xfam.org/search) [3]. Conserved motif were predicted using the MEME Suite with the default parameter (https://meme-suite.org/meme/) [4].

Table S8. Forward (*F*) and reverse (*R*) primer sequences used in real-time quantitative PCR

Gene	Primer sequences (5′→3′)	Gene ID
ZmSTRL1	F: CAGCTGGCCCAGAGACAATG<br>R: ATTCATGACGGCGAACGTCT	Zm00001d047858
ZmSTRL2	F: CTTCGGAGGGTGTTCAGGTC<br>R: TGTCCATCGACGACACAAGG	Zm00001d052471
ZmSTRL3	F: CAGTTGTGTTGCGTGTGCAT<br>R: ACCATTCCACGAACGGAGTC	Zm00001d020306

ZmSTRL4	F: GTAGGGGCTGCAGGTTCCA<br>R: CAAAGCGAGGAGCCACGTAA	Zm00001d012371
ZmSTRL8	F: TTTTGAGGGTCGGTGACGAG<br>R: CGGTGGAGCTGAACTCTAGG	Zm00001d006853
ZmSTRL13	F: GGATCGGACATCGGAGTTGT<br>R: AGTGCTCTCCTCCCGATGAT	Zm00001d012249
ZmSTRL14	F: GGGAAGCTCTGGTTAGGCTC<br>R: TGGGTTGCCAAACTAGCCC	Zm00001d012375
ZmSTRL19	F: TACCGAAAGCAAGCAAGGCT<br>R: GGCCCACGTAGATGGACTTC	Zm00001d020308

1. Lescot, M.; Déhais, P.; Thijs, G.; Marchal, K.; Moreau, Y.; Van de Peer, Y.; Rouzé, P.; Rombauts, S. PlantCARE, a database of plant cis-acting regulatory elements and a portal to tools for in silico analysis of promoter sequences. *Nucleic Acids Research* **2002**, *30*, 325-327, doi:10.1093/nar/30.1.325.
2. Subramanian, B.; Gao, S.; Lercher, M.J.; Hu, S.; Chen, W.-H. Evolview v3: a webserver for visualization, annotation, and management of phylogenetic trees. *Nucleic Acids Research* **2019**, *47*, W270-W275, doi:10.1093/nar/gkz357.
3. Paysan-Lafosse, T.; Blum, M.; Chuguransky, S.; Grego, T.; Pinto, B.L.; Salazar, Gustavo A.; Bileschi, Maxwell L.; Bork, P.; Bridge, A.; Colwell, L.; et al. InterPro in 2022. *Nucleic Acids Research* **2023**, *51*, D418-D427, doi:10.1093/nar/gkac993.
4. Grant, C.; Bailey, T. *XSTREME: Comprehensive motif analysis of biological sequence datasets*; 2021.
